# Simple aneuploidy evades p53 surveillance and promotes niche factor-independent growth in human intestinal organoids

**DOI:** 10.1091/mbc.E24-04-0166

**Published:** 2024-07-22

**Authors:** Blake A. Johnson, Albert Z. Liu, Tianhao Bi, Yi Dong, Taibo Li, Dingjingyu Zhou, Akshay Narkar, Yufei Wu, Sean X. Sun, Tatianna C. Larman, Jin Zhu, Rong Li

**Affiliations:** a Department of Cell Biology, Johns Hopkins School of Medicine, Baltimore, MD 21205; e Department of Pathology, Division of Gastrointestinal/Liver Pathology, Johns Hopkins School of Medicine, Baltimore, MD 21205; b Department of Biomedical Engineering, Johns Hopkins University, Baltimore, MD 21218; c Department of Mechanical Engineering, Johns Hopkins University, Baltimore, MD 21218; d Institute for NanoBio Technology, Johns Hopkins University, Baltimore, MD 21218; f Mechanobiology Institute, National University of Singapore, Singapore 117411, Singapore; g Department of Biological Sciences, National University of Singapore, Singapore 117558, Singapore; University of North Carolina at Chapel Hill

## Abstract

Aneuploidy is nearly ubiquitous in tumor genomes, but the role of aneuploidy in the early stages of cancer evolution remains unclear. Here, by inducing heterogeneous aneuploidy in non-transformed human colon organoids (colonoids), we investigated how the effects of aneuploidy on cell growth and differentiation may promote malignant transformation. Previous work implicated p53 activation as a downstream response to aneuploidy induction. We found that simple aneuploidy, characterized by 1–3 gained or lost chromosomes, resulted in little or modest p53 activation and cell cycle arrest when compared with more complex aneuploid cells. Single-cell RNA sequencing analysis revealed that the degree of p53 activation was strongly correlated with karyotype complexity. Single-cell tracking showed that cells could continue to divide despite the observation of one to a few lagging chromosomes. Unexpectedly, colonoids with simple aneuploidy exhibited impaired differentiation after niche factor withdrawal. These findings demonstrate that simple aneuploid cells can escape p53 surveillance and may contribute to niche factor-independent growth of cancer-initiating colon stem cells.

## Abstract (executive summary)

Aneuploidy is common in colon cancer, yet the consequences of aneuploid cells in the normal colon epithelium are not well understood.

Using colon organoids to model the human intestine, the authors found that complex aneuploid cells activated p53 and arrested while simple aneuploid cells often continued to divide and failed to terminally differentiate.

These findings suggest simple aneuploid cells can escape p53 surveillance and continue to proliferate even when growth factors are not present, which may contribute to early colon cancer evovlution.

## INTRODUCTION

Aneuploidy, which arises due to chromosome missegregation in mitosis, is prevalent in cancer, with up to 90% of solid tumor genomes harboring aberrant karyotypes, but is uncommon in healthy tissues (Knouse *et al.*, 2014; Taylor *et al.*, 2018). It remains unclear how aneuploidy may be either eliminated from or tolerated in proliferating tissues and how aneuploidy may contribute to the initiation of neoplasia. Early studies suggested that aneuploidy activates the tumor suppressor p53, thereby preventing further expansion of cells with altered karyotypes (Li *et al.*, 2010; Thompson and Compton, 2010; Foijer *et al.*, 2014). However, more recent studies have revealed that not all aneuploid cells activate p53 (Santaguida *et al.*, 2017; Soto *et al.*, 2017; Garribba *et al.*, 2023). In our previous work, we demonstrated that p53 activation is less robust in aneuploid mouse colon and human mammary organoids when compared with certain cancer and immortalized cell lines, pointing to the possible importance of tissue context or cell-type effects in the aneuploidy response (Narkar *et al.*, 2021). This finding prompted us to further investigate the consequences of aneuploidy induction within nontransformed tissues in this study.

Colon organoids (colonoids), which are derived from intestinal stem cells, have been utilized to model tumor evolution (Drost *et al.*, 2015; Matano *et al.*, 2015; Fujii *et al.*, 2016; Fumagalli *et al.*, 2017; Bolhaqueiro *et al.*, 2019; Lannagan *et al.*, 2019). For example, cancer driver mutations were introduced in colonoids to elucidate their individual contributions to niche factor-independent growth (Drost *et al.*, 2015; Matano *et al.*, 2015), and colonoids derived from colorectal cancers were used to demonstrate that continual chromosomal instability (CIN) generates novel aneuploid karyotypes and shapes genome evolution in colorectal cancer (Bolhaqueiro *et al.*, 2019).

In this study, we derived colonoids from nontransformed human colon mucosa to assess the effects of aneuploidy on the fate of intestinal stem cells and the possible contribution of aneuploidy towards early tumorigenesis. Although our data confirm that p53 activation is indeed a common response to aneuploidy, we demonstrate that aneuploid cells with simple karyotypes continue to proliferate even in the absence of niche factors and may thus contribute to the early genetic diversity that enables cancer evolution.

## Results and Discussion

### Differential proliferative capacity of simple and complex aneuploid cells

**FIGURE 1.**
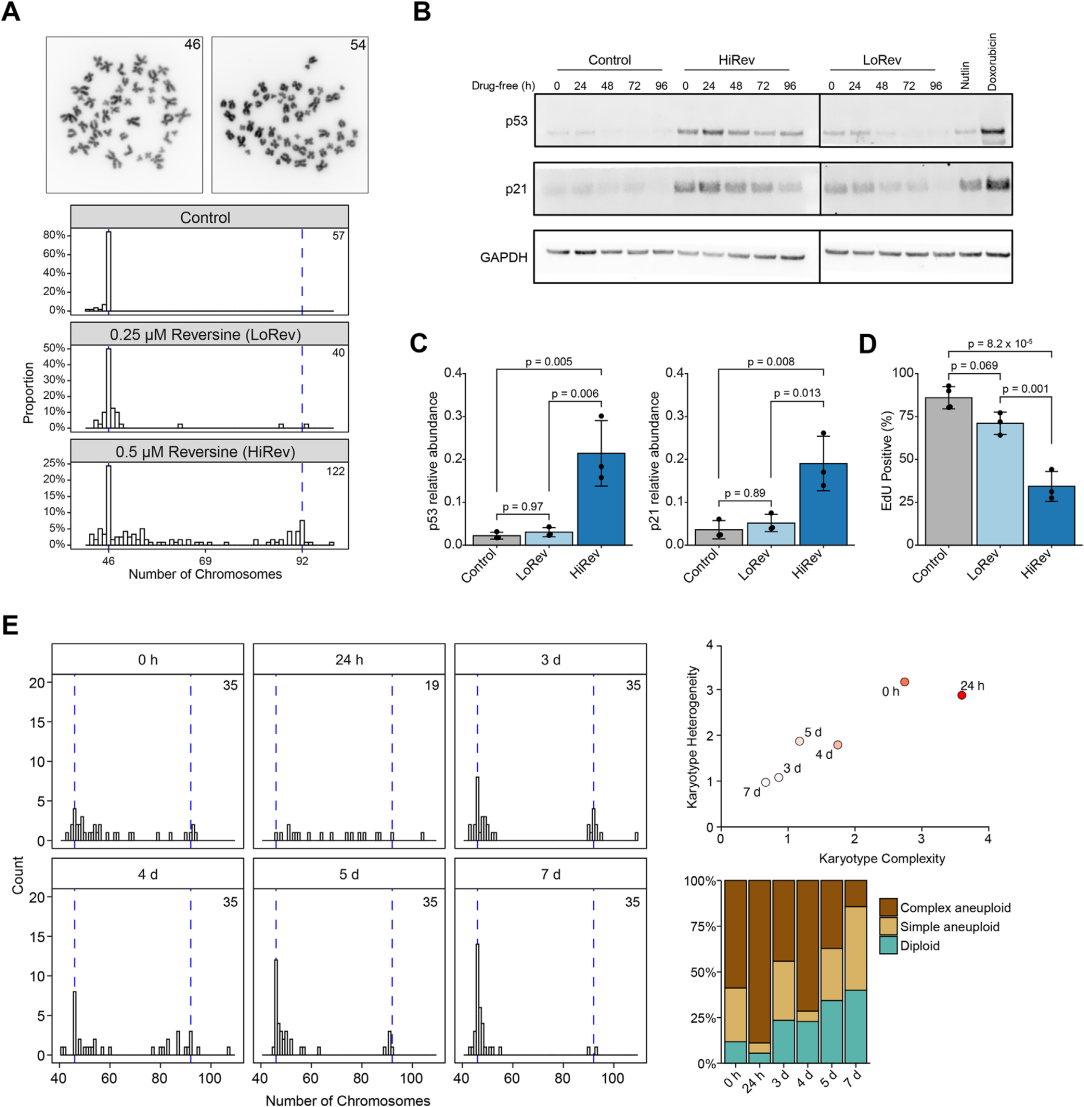
Differential proliferative capacity of simple and complex aneuploid cells. (A) (top) Representative images of metaphase spread from colonoids treated with vehicle-control (left) and 0.5 µM reversine (right). (bottom) Karyotype distributions assessed by metaphase spreads of colonoids treated with vehicle-control (DMSO), 0.25 µM reversine (LoRev), or 0.5 µM reversine (HiRev) for 24 h followed by 24 h in drug-free proliferation media. *n* = 5 independent experiments, a number of metaphase spreads shown in the upper right. (B) Immunoblot of human colonoids treated with DMSO (vehicle-control) or reversine for 24 h followed by 0, 24, 48, 72, or 96 h in drug-free proliferation media. Quantification of 24 h drug-free timepoint is shown in Figure 1C. GAPDH is a loading control. Membrane was cut before antibody staining and cut edges are visible in the presented images. Two membranes were needed due to the number of samples in this experiment. Separate membranes are denoted by a vertical black line. (C) p53 and p21 quantified by Western blot in colonoids treated for 24 h followed by 24 h in drug-free proliferation media. Relative abundance to GAPDH, loading control. *n* = 3 biological replicates. Bars represent mean and error bars represent SD. Abundances compared using ANOVA and posthoc Tukey test. Tukey *p* values are shown. (D) Percentage of EdU positive cells quantified by flow cytometry. Conditions as in panel C. *n* = 3 biological replicates. Plot elements and statistics as in panel C. (E) (left) Karyotype distributions, (right, top) karyotype complexity and heterogeneity, and (right, bottom) percentage of diploid, simple aneuploid, or complex aneuploid cells assessed by metaphase spreads of colonoids treated with HiRev for 24 h followed by 0 h, 24 h, 3 d, 4 d, 5 d, or 7 d in drug-free proliferation media. Number of metaphase spreads shown in top right on each histogram.

We generated nontransformed colonoids from histologically normal mucosa of a surgically resected CRC specimen (Supplemental Figure S1A). Colonoids grew at the expected rate and with similar morphology to previous reports (Sato *et al.*, 2011). Also, p21 abundance increased, and colonoids failed to expand upon administration of Nutlin-3a, confirming that p53 was functional in our colonoid line (Supplemental Figure S1, B and C). To generate heterogenous aneuploid cell populations, we treated colonoids with numerous antimitotic drugs, which led to a range of aneuploid karyotypes (Supplemental Figure S1D). Treatment with a lower concentration of reversine (0.25 µM, LoRev), an MPS1 kinase inhibitor (Santaguida *et al.*, 2010), induced aneuploidy in 50% of cells with the vast majority having 1–3 aneuploid chromosomes, whereas treatment with a higher concentration (0.5 µM, HiRev), induced a wider range of karyotypes in over 75% of cells (Figure 1A). We grouped aneuploid cells into two categories based on the karyotypes generated by these conditions: simple (1–3 chromosome changes) and complex (≥4 chromosomes change). Live imaging confirmed that reversine accelerated mitosis (Supplemental Figure S1E). Lagging chromosomes were the predominant mitotic abnormalities observed in LoRev colonoids (Supplemental Figure S1F). We examined p53 and p21 abundance over time following aneuploidy induction (Figure 1B; Supplemental Figure S1G). LoRev colonoids did not demonstrate an increased abundance of p53 or p21 at 24 h after reversine withdrawal, whereas both proteins were increased in HiRev colonoids (Figure 1C). LoRev and HiRev colonoids had different proportions of aneuploid cells (50 and 76%, respectively), but p53 abundance in the HiRev colonoids still exceeded that of LoRev following normalization for the increased prevalence of aneuploidy (Supplemental Figure S1H). In addition, we observed a similar pattern of p53 activation related to karyotype complexity following treatment with low and high concentrations of NMS-P715, an alternative MPS1 kinase inhibitor (Colombo *et al.*, 2010) (Supplemental Figure S1I). LoRev colonoids proliferated comparably to vehicle-control as shown by similar EdU incorporation (Salic and Mitchison, 2008), although we did observe a trend toward reduced proliferation (Figure 1D). HiRev colonoids had significantly reduced proliferation (Figure 1D). As HiRev generated a heterogenous aneuploid population that included both simple and complex aneuploidy, we used this population to observe how karyotype complexity evolved over time. Consistent with the modest p53 activation of LoRev colonoids, the simple aneuploid subpopulation of HiRev colonoids expanded while the complex aneuploid population was eliminated over 7 d (Figure 1E). Together, these data suggest that many, but perhaps not all, simple aneuploid cells can evade p53 surveillance and continue to proliferate. Importantly, karyotyping by metaphase spreads is error-prone and can only assess dividing cells, which could explain the unexpectedly high proportion of aneuploid cells in control colonoids (Figure 1A). Therefore, we proceeded to utilize single-cell RNA sequencing to further investigate these findings.

### p53-driven gene expression correlates with karyotype complexity

**FIGURE 2.**
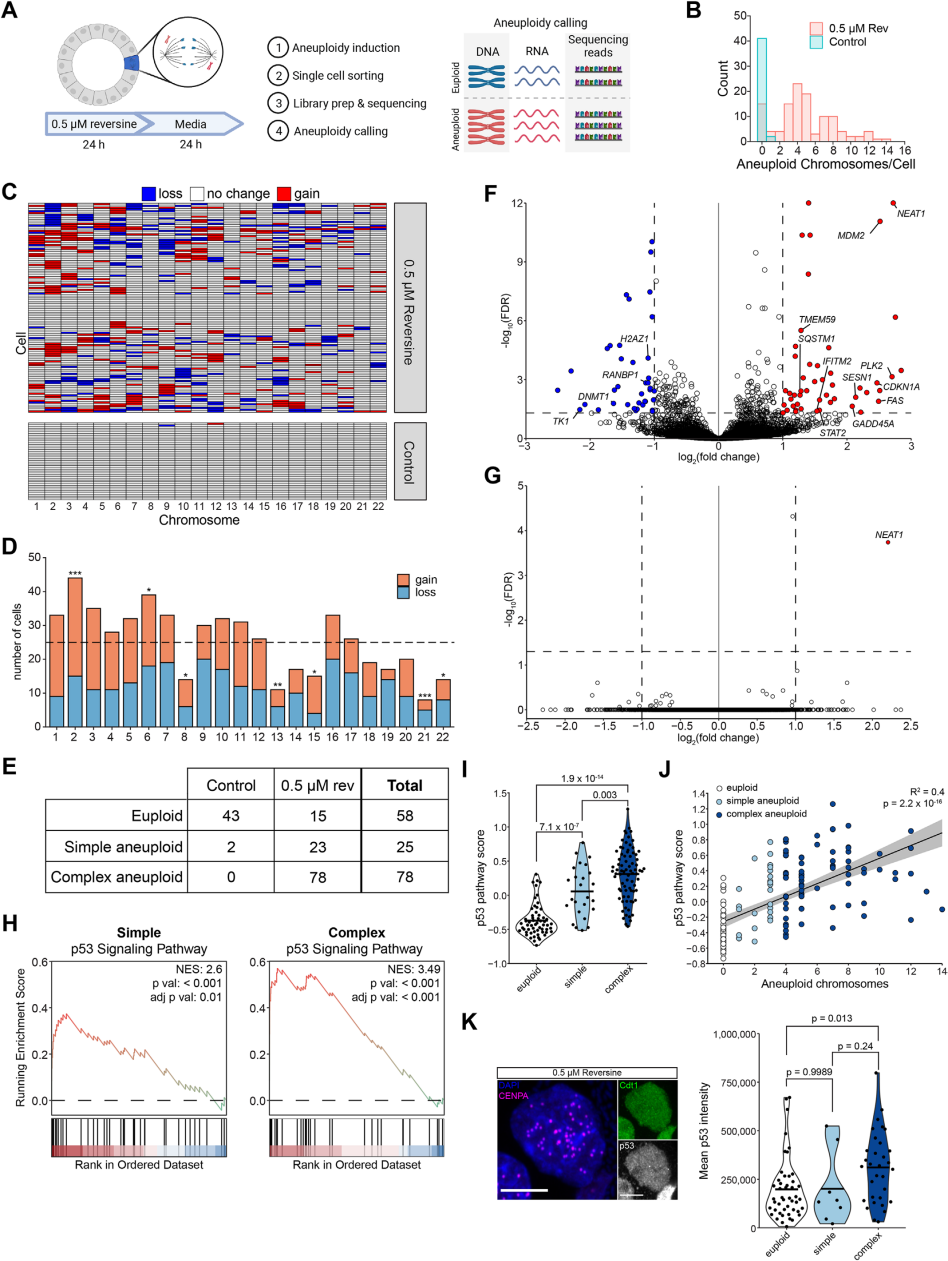
p53-driven gene expression correlates with karyotype complexity. (A) Schematic of single-cell RNA sequencing (scRNAseq) of aneuploid cells in colonoids. (B) Distribution of aneuploid chromosomes per cell from scRNAseq. Each chromosome gain or loss is defined as 1 aneuploid chromosome. (C) Karyotypes of 116 cells treated with 0.5 µM reversine and 45 vehicle-control cells quantified from scRNAseq. Cells were grouped by hierarchical clustering. (D) Bar plot showing the frequency at which each chromosome was gained or lost in all cells treated with 0.5 µM reversine quantified by single-cell RNA sequencing. Dashed line represents expected frequency of 25 aneuploid events per chromosome based on a binomial (n, P) distribution with *n* = 116 and *P* = 0.21826. * *p* < 0.05, ** *p* < 0.01, *** *p* < 0.001. (E) Table showing number of cells in each group included in scRNA-seq analysis. (F) Differentially expressed genes comparing cells with ≥4 aneuploid chromosomes (complex) and euploid cells. Cutoffs for differential expression were log_2_(fold change) >1 or <–1 (shown by vertical dashed lines) and FDR ≤ 0.5 (shown by horizontal dashed line). Genes with positive log_2_ (fold change) are more highly expressed in aneuploid cells. (G) Differentially expressed genes comparing cells with 1–3 aneuploid chromosomes (simple) and euploid cells. Features are the same as Panel F. (H) Gene set enrichment analysis plots demonstrating enrichment of genes in the p53 signaling pathway in aneuploid cells. NES: normalized enrichment score. (I) Violin plot showing p53 pathway score in euploid, simple aneuploid, and complex aneuploid cells from scRNA-seq. Dots represent individual cells, and the bar represents the mean. Scores compared using ANOVA and posthoc Tukey test. Tukey *p* values are shown. (J) Relationship between p53 pathway expression and the number of aneuploid chromosomes in cells treated with 0.5 µM reversine in scRNAseq data. Line shows linear regression. (K) (left) Representative image of a fixed cell expressing Cdt1-eGFP-T2A-CENPA-Halo and immunostained for p53. The HaloTag ligand JF549 was added. Scale bar = 5 µm. (right) Violin plot showing mean p53 intensity in nuclei from euploid, simple aneuploid, and complex aneuploid cells from Cdt1-eGFP-T2A-CENPA-Halo + p53 immunofluorescence imaging. ≤ 47 chromosomes = euploid, 48 or 49 chromosomes = simple aneuploid, ≥50 chromosomes = complex aneuploid. *n* = 3 independent experiments. Plot features and stats as in panel I. Rev = reversine, Control = 0.05% DMSO

To investigate the transcriptional consequences of both simple and complex aneuploidy, we performed high-depth, full-length single-cell RNA seq (scRNAseq) of cells from colonoids after treatment with HiRev (Figure 2A). As the average abundance of RNA transcripts across a chromosome scales with changes in the copy number of the chromosome (Li and Zhu, 2022), scRNAseq data can also be used to infer alterations in chromosome stoichiometry (Patel *et al.*, 2014; Griffiths *et al.*, 2017; Serin Harmanci *et al.*, 2020; Gao *et al.*, 2021). Using this approach (see *Materials and Methods*), we detected many aneuploid cells in colonoids treated with reversine, while few aneuploid cells were detected within control colonoids (Figure 2B). It is important to note that our analysis was designed to identify whole chromosome, not segmental, changes in copy number. The observed aneuploid karyotypes were heterogenous, displaying both chromosome gains and losses across the genome (Figure 2C). Consistent with previous reports (Worrall *et al.*, 2018; Klaasen *et al.*, 2022), larger chromosomes were more likely to be missegregated (Figure 2D).

We compared the transcriptomes of simple and complex aneuploid cells to euploid controls (Figure 2E). UMAP revealed that all cells in the analysis separated into two clusters (Supplemental Figure S2A). This clustering appeared to be largely driven by differences in the expression of cell-cycle related genes (Supplemental Figure S2B). Differential expression analysis revealed increased expression of p53 responsive genes, such as *NEAT1*, *MDM2*, *PLK2*, *CDKN1A*, *FAS*, and *GADD45A,* in cells with complex aneuploidy compared with diploid cells (Figure 2F; Supplemental Table 1). On the other hand, comparing the simple aneuploid population with diploid yielded only one of the above differentially expressed genes, *NEAT1* (Figure 2G; Supplemental Table 1). Gene set enrichment analysis also revealed strong enrichment of genes related to the p53 signaling pathway in complex aneuploid cells, with weaker enrichment observed for simple aneuploidy (Figure 2H; Supplemental Figure S2, C and D).

We further observed that the p53 pathway expression score (Tirosh *et al.*, 2016) in individual cells was increased in simple aneuploid cells compared with control and further increased in complex aneuploid cells (Figure 2I). p53 pathway expression positively correlated with the number of aneuploid chromosomes (Figure 2J). Increased p53 pathway expression was not associated with gain or loss of chromosome 17, which carries the *TP53* gene (Supplemental Figure S2E). To directly visualize the relationship between chromosome number and p53 abundance, we generated colonoids expressing CENPA-Halo, a centromere marker (Schalch and Steiner, 2017), and eGFP-Cdt1, which allows visualization of G1 cells (Sakaue-Sawano *et al.*, 2008; Figure 2K). Using quantitative analysis to resolve clustered CENPA foci in G1 cells (see *Materials and Methods*), we identified cells with complex aneuploidy (Supplemental Figure S2F). However, we were unable to reliably identify cells that gained or lost a small number of chromosomes using this method, so cells with ≤47 CENPA foci were considered euploid in this analysis. Colonoids treated with HiRev had more cells with increased p53 fluorescence intensity when compared with control or LoRev (Supplemental Figure S2G). p53 intensity was increased in complex aneuploid cells compared with euploid cells (Figure 2K). These results demonstrate that p53 activation in aneuploid cells is positively correlated with karyotype complexity.

### p53 activation in aneuploid populations is not driven by DNA damage

DNA damage, which may result from damage during chromosome missegregation or replication stress, has been proposed to lead to p53 activation in aneuploid cells (Janssen *et al.*, 2011; Santaguida *et al.*, 2017; Soto *et al.*, 2017; Gemble *et al.*, 2022; Garribba *et al.*, 2023). Cells in HiRev colonoids with increased p53 activity rarely had cooccurring increased pH2AX, a marker of DNA damage (Mah *et al.*, 2010; Supplemental Figure S3A). In colonoids expressing H2B-mNeon and mCherry-53BP1, DNA damage foci were infrequently observed in the 8 h immediately following chromosome missegregation suggesting against direct DNA damage during erroneous mitosis (Supplemental Figure S3B). To assess whether replication stress in S phase could cause DNA damage and lead to p53 activation in aneuploid cells, we treated colonoids with 0.25 or 0.5 µM reversine for 4 h, followed by 16 h in drug-free media to allow cells to progress through S phase. We did not observe any difference in the mean pH2AX intensity between cells with or without increased p53 abundance, suggesting that DNA damage could not explain the observed difference in p53 (Supplemental Figure S3C). We also blocked the DNA damage response both during and after aneuploidy induction using inhibitors of ATM and ATR, key kinases which link DNA damage signaling to p53 activation (Maréchal and Zou, 2013). We observed no change in p53 or p21 abundance and a minute, though statistically significant, increase in EdU incorporation only after treatment with both inhibitors for 48 h (Supplemental Figure S3, D and E). These findings suggest DNA damage is not the major driver of p53 activation in complex aneuploid cells.

### Direct tracking of proliferative cell fate following chromosome missegregation

**FIGURE 3.**
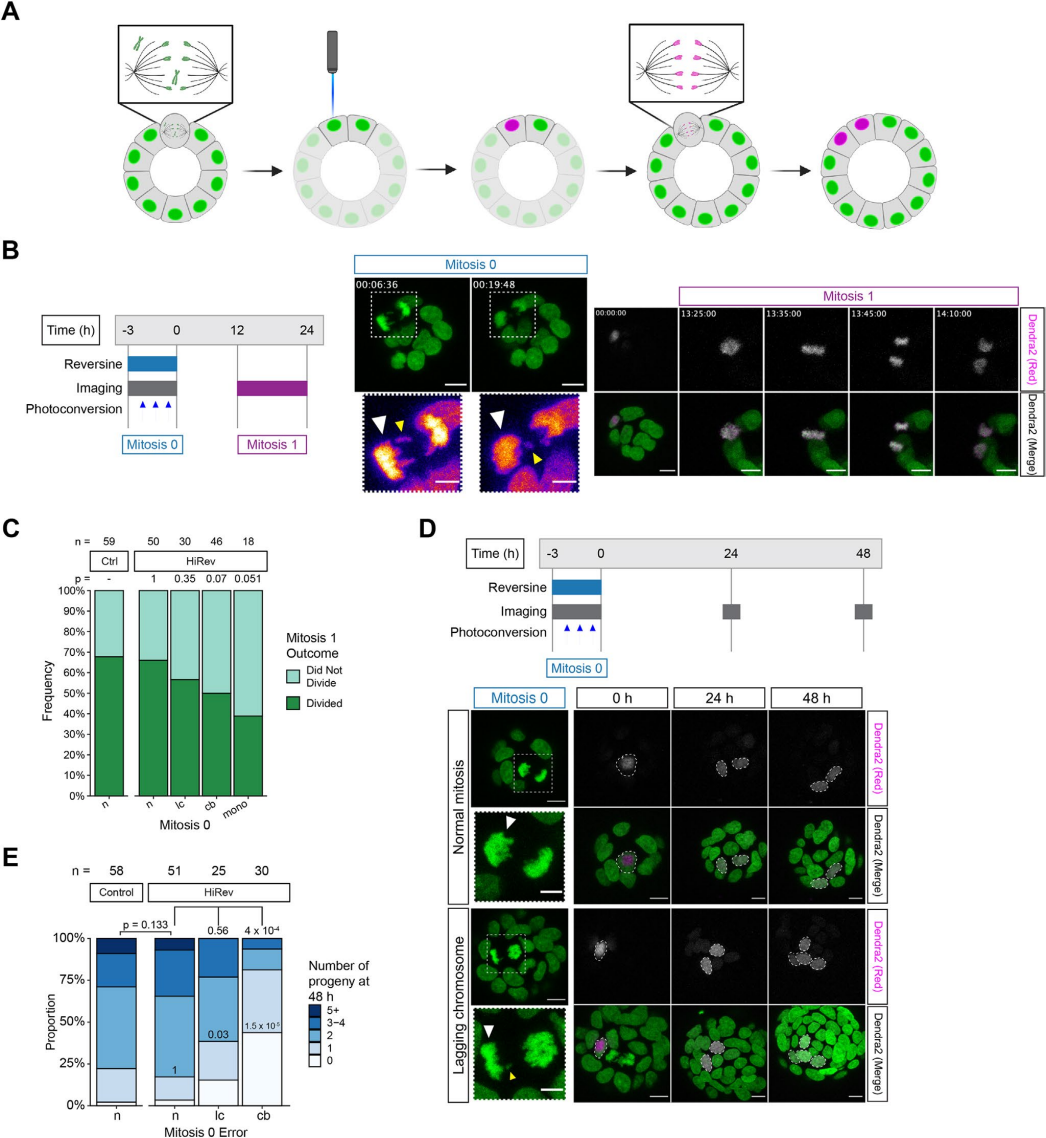
Direct tracking of proliferative cell fate following chromosome mis-segregation. (A) Schematic representation of H2B-Dendra2 photoconversion experiment. (B) (left) Schematic representation of experiment timeline. (right) Representative images of a photoconverted cell dividing the following division with a lagging chromosome. The white arrowhead denotes a photoconverted cell. Yellow arrowheads denote a lagging chromosome. Scale bar = 10 µm, inset scale bar = 5 µm. (C) Division frequency of photoconverted cells in Mitosis 1. *n* ≥ 5 biological replicates. The total number of cells tracked is shown above each bar. *p* values calculated using Fisher’s exact test comparing the condition of interest to control. (D) (top) Schematic representation of experiment. (bottom) Representative images of photoconverted cells. Normal mitosis (top panel) was treated with 0.05% DMSO and the lagging chromosome (bottom panel) was treated with 0.5 µM reversine. White arrowheads denote the photoconverted cell. The yellow arrowhead denotes a lagging chromosome. Scale bar = 10 µm, inset scale bar = 5 µm. (E) Number of progeny 48 h after Mitosis 0. *n* = 5 biological replicates. The total number of cells tracked is shown above each bar. *p* values were calculated using Fisher’s exact test. *p* values above the bar compare the entire progeny distribution. *p* values shown within the bar compare cells with 0 or 1 progeny of each group to control. HiRev = 0.5 µM reversine, *n* = normal, lc = lagging chromosome, cb = chromatin bridge, mono = monopolar spindle, control = 0.05% DMSO.

To directly observe the proliferative fate of individual aneuploid cells, we generated colonoids expressing H2B-Dendra2, a photoconvertible fluorescent protein (FP), and converted one of the daughter nuclei from green to red fluorescence following chromosome missegregation as previously described (Bollen *et al.*, 2021; Figure 3, A and B Mitosis 0). This was followed by time-lapse imaging to determine whether the photoconverted cell progressed through the subsequent cell cycle (Figure 3B Mitosis 1, Supplemental Video S1). HiRev was used in these experiments because it promotes more chromosome missegregation than LoRev. We observed that daughter cells from divisions with lagging chromosomes in Mitosis 0 divided again in Mitosis 1 nearly as frequently as daughter cells from normal divisions (Figure 3C). The presence or absence of a micronucleus did not impact the division frequency (Supplemental Figure S4A). An important caveat of this experiment is that we were unable to determine the specific karyotype of the cells we tracked. However, we observed few (≤3) lagging chromosomes in each division, and previous work has demonstrated divisions with lagging chromosomes frequently lead to simple aneuploid karyotypes (Bollen *et al.*, 2021). Therefore, the daughter cells we tracked were likely to be simple aneuploids. Daughter cells from divisions with chromatin bridges or monopolar spindles in Mitosis 0 were less likely to divide in Mitosis 1 than cells born from divisions with lagging chromosomes (Figure 3C). DNA damage in mitosis can lead to telomere fusion, resulting in chromatin bridges (Deckbar *et al.*, 2007; Orthwein *et al.*, 2014). 53BP1 foci were not increased after 4 h of imaging or before chromatin bridge formation, suggesting imaging-related phototoxicity did not promote this type of error (Supplemental Figure S4B). For those cells that proceeded to Mitosis 1, none of the error types observed in Mitosis 0 delayed cell cycle progression (Supplemental Figure S4C).

**Figure:** Movie S1 **Representative time‐lapse H2B‐10 Dendra2 tracking movie demonstrating division after chromosome mis‐segregation, related to Figure 3**. Mitosis 0 time interval: 3 minutes 18 seconds, Mitosis 1 time interval: 5 minutes. Mitosis 1 imaging started 12 hours after mitosis 0. Non‐photoconverted H2B‐Dendra2 shown in green. Photoconverted H2B‐Dendra2 shown in magenta. Mitosis 1 images were zoomed in to the photoconverted cell at the time of imaging.

We next quantified the number of progeny arising from a single photoconverted cell 48 h (about two cell cycles) after chromosome missegregation (Figure 3D). The original photoconverted cell was included in this quantification. Zero (0) progeny were recorded if the original cell had died, and one progeny indicated likely cellular arrest. Daughter cells arising from normal divisions or divisions with lagging chromosomes in Mitosis 0 had a similar overall number of progeny (Figure 3E). However, lagging chromosomes led to more colonoids with 0 or 1 photoconverted progeny cells, suggesting that these cells are more likely to fail to divide or die (Figure 3E; Supplemental Figure S4D). While a portion of cells arising from divisions with chromatin bridges divided in Mitosis 1, most either arrested or died at the 48 h timepoint (Figure 3E). These cells were frequently binucleate in Mitosis 1 and often underwent multipolar division, which likely resulted in complex aneuploidy, leading to cell cycle arrest or death at this timepoint (Supplemental Figure S4, D and E). These observations suggest that many, but not all, cells continue to proliferate following chromosome missegregation.

### Intestinal stem cells with simple aneuploidy exhibit impaired differentiation

**FIGURE 4.**
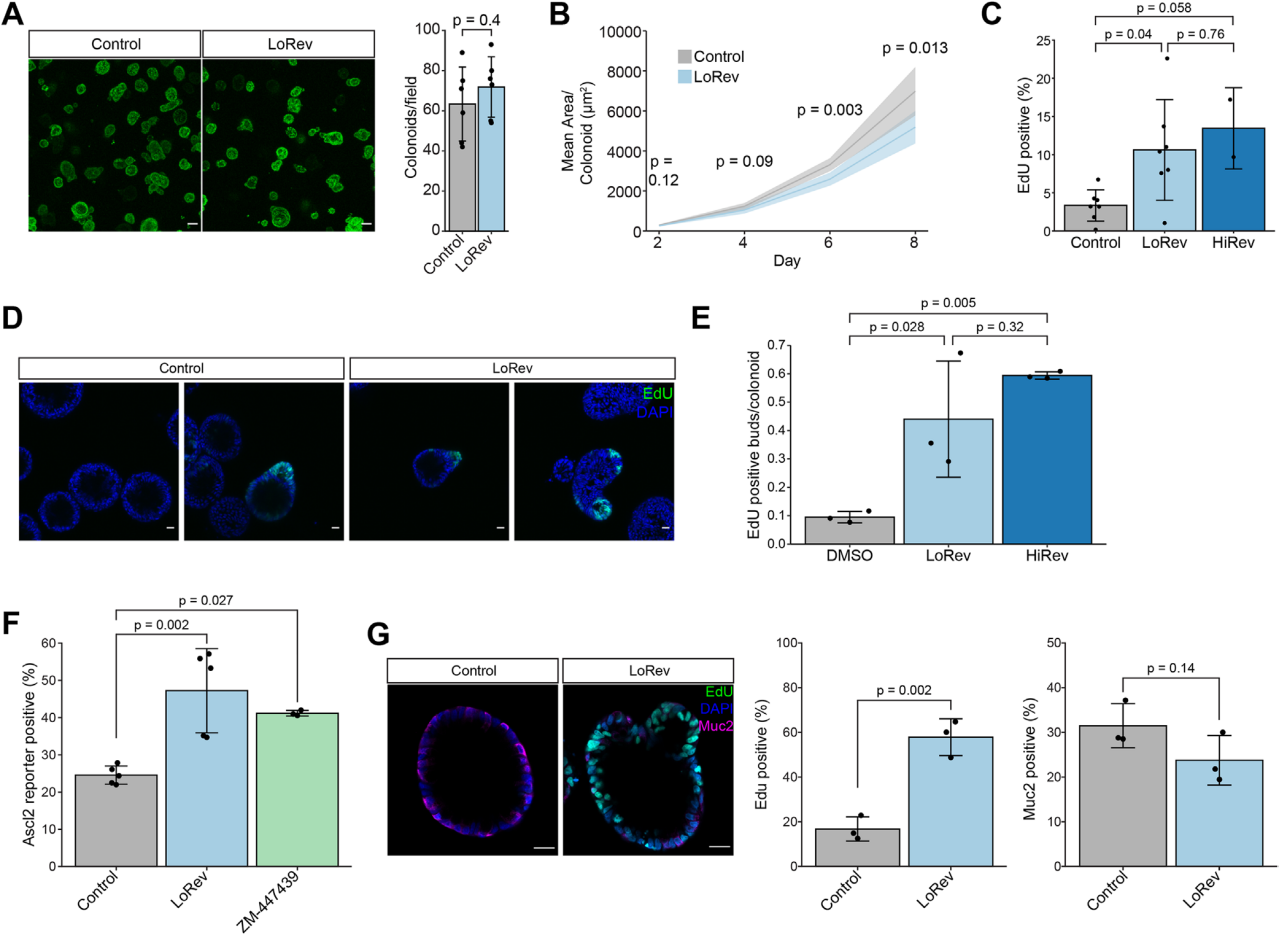
Intestinal stem cells with simple aneuploidy exhibit impaired differentiation. (A) (left) Representative images of H2B-Dendra2 colonoids formed from single cells 8 d after plating. (right) Quantification of the colonoid forming efficiency. *n* = 6 biological replicates. *p* value was calculated by two-sided Welch’s two-sample *t* test. Scale bar = 100 µm. (B) Mean colonoid area over time of colonoids formed from single cells. The area was quantified from maximum intensity projections. *n* = 6 biological replicates. *p* values were calculated by two-sided Welch’s two-sample *t* test. Ribbon shows SD. (C) EdU positive cells in LoRev colonoids treated for 24 h followed by 96 h in drug-free differentiation media measured by flow cytometry. EdU was added to differentiation media for 16 h. *n* = 5 biological replicates for control and LoRev and 2 for HiRev. Compared using ANOVA and posthoc Tukey test. Tukey *p* values are shown. (D) Representative images of EdU localization in colonoid buds after 96 h of differentiation. EdU was added to differentiation media for 16 h. Scale bar = 20 µm. (E) Number of EdU positive buds per colonoid from images as in Panel D. *n* = 3 biological replicates. Compared using ANOVA and posthoc Tukey test. Tukey *p* values are shown. (F) *ASCL2* reporter positive cells in LoRev and ZM-447439 colonoids following 96 h in drug-free differentiation media measured by flow cytometry. *n* ≥ 3 biological replicates. Compared using ANOVA and posthoc Tukey test. Tukey *p* values are shown. (G) (left) Representative images of colonoids labeled with EdU and immunostained for Muc2 following 96 h of differentiation. (right) Quantification of EdU and Muc2 positive cells. *n* = 3 biological replicates. *p* value was calculated using a two-sided two-sample *t* test. Bars represent mean, and error bars represent SD throughout the figure. Control = 0.05% DMSO

As simple aneuploid cells could remain proliferative, we examined their stem cell capacity and ability to form colonoids from single cells. LoRev colonoids grown in proliferation media retained expression of stem cell marker genes at the same level as control colonoids (Supplemental Figure S5A). We found no difference in organoid-forming frequency between LoRev and vehicle-control colonoids following dissociation to single cells (Figure 4A). However, colonoids generated from LoRev single cells grew more slowly in proliferation media (Figure 4B).

We next assessed the ability of aneuploid stem cells to differentiate by removing stem cell growth factors and differentiation inhibitors from the culture media (Sato *et al.*, 2011). Surprisingly, cells in LoRev and HiRev colonoids were more likely to incorporate EdU, indicating proliferation, following 96 h of differentiation compared with diploid controls, though the difference was only statistically significant for LoRev (Figure 4C). We did not observe higher expression of stem cell marker genes at the population level, as measured by RT-qPCR, in LoRev colonoids (Supplemental Figure S5B), suggesting that the impaired differentiation likely occurred in only a subset of the cells. Consistently, whereas the control diploid colonoids became uniformly spherical following differentiation, similar to previously observed phenotypes (Lukonin *et al.*, 2020), LoRev colonoids contained crypt-like buds (Supplemental Figure S5C). These buds frequently contained proliferative (EdU positive) cells (Figure 4D), consistent with a previous study demonstrating that buds contain stem cells in intestinal organoids (Serra *et al.*, 2019). Furthermore, EdU positive buds were more prevalent in LoRev and HiRev colonoids compared with diploid control (Figure 4E). While HiRev did not result in a statistically significant increase in impairment of differentiation compared with LoRev, there was a trend in this direction. Impaired differentiation was not explained by the slow growth of aneuploid cells, as treatment of colonoids with low concentrations of Nutlin-3a or Palbociclib to slow down, but not arrest, growth did not have the same effect as reversine (Supplemental Figure S5, D and E). LoRev colonoids also had increased expression of an *ASCL2* reporter, which marks intestinal stem cells (Oost *et al.*, 2018) following differentiation (Figure 4F). Treatment with the Aurora B kinase inhibitor ZM-447439 (Ditchfield *et al.*, 2003), which leads to similar karyotype complexity and heterogeneity to LoRev (Supplemental Figure S1D), also resulted in increased *ASCL2* expression following differentiation (Figure 4F). LoRev colonoids had a trend toward reduced expression of Muc2, a marker for differentiated goblet cells, but this difference was not statistically significant (Figure 4G). Together, these findings suggest that simple aneuploid stem cells not only continue to proliferate but also show impaired differentiation in the absence of stem cell niche factors.

Previous studies using different experimental models showed that the fate of aneuploid cells, especially p53 activation and cell cycle arrest, is varied and may depend on cellular and physiological contexts (Gogendeau *et al.*, 2015; Resende *et al.*, 2018; Ben-David and Amon, 2020; Hoevenaar *et al.*, 2020; Vasudevan *et al.*, 2021). Here, we investigated the effect of aneuploidy within the normal colon epithelium using patient-derived human colonoids. Using high-depth, full-length single-cell RNA sequencing, biochemical assays, and live-cell imaging approaches, our data suggest that the degree of aneuploidy plays a crucial role in determining how cells respond. In agreement with previous studies (Thompson and Compton, 2010; Santaguida *et al.*, 2017; Soto *et al.*, 2017), we found that complex aneuploid cells activate p53 and are eliminated from the population. In contrast, simple aneuploid cells generated with LoRev treatment had less effect on p53 activity. Our data suggest that simple aneuploid cells can mildly activate the p53 pathway in human colonoids, particularly at the transcriptional level, and this effect is markedly enhanced as karyotype complexity increases. Importantly, our data also suggest that at least a subset of aneuploid cells gaining or losing one or a few chromosomes can evade p53 surveillance and continue to proliferate. This contrasts a previous finding in RPE-1 cells (Hinchcliffe *et al.*, 2016) and may highlight a key difference in the response to aneuploidy between cells in the context of three-dimensional organoids and two-dimensional cultured cell lines. Nonetheless, our study also confirmed that p53 does respond to high-level (complex) aneuploidy and plays a role in its elimination from epithelial tissues.

The impact of aneuploidy on differentiation has been investigated in both embryonic and adult stem cells in a variety of organisms (Ben-David *et al.*, 2014; Gogendeau *et al.*, 2015; Pfau *et al.*, 2016; Zhang *et al.*, 2016; Resende *et al.*, 2018). Our data suggest that aneuploidy impairs differentiation of human colon stem cells, which are the cell of origin in colorectal cancer (Barker *et al.*, 2009). Our finding is consistent with the observation that aneuploidy resulted in significant expansion of the proliferative zone in intestinal crypts in a mouse model of chromosomal instability (Hoevenaar *et al.*, 2020). In our study, only a small percentage of cells treated with LoRev retained proliferative capacity upon niche factor withdrawal. There are two potential explanations. First, the observed proliferation may be a karyotype-specific phenotype resulting from the gain or loss of a particular chromosome or set of chromosomes. Second, aneuploidy may generally decrease the probability of differentiation, resulting in more proliferative cells. Our analysis examined only bulk populations, including both simple aneuploid and euploid cells. Isolating single proliferative cells under differentiation conditions and studying their karyotype and stem cell potential will help establish a causal role for aneuploidy in impairing colon stem cell differentiation. Although further studies are necessary to elucidate the underlying mechanism, our finding sheds light on one possible role for aneuploidy in the initiation of tumorigenesis, as niche-factor independent growth is an important early step in CRC evolution (Drost *et al.*, 2015).

## MATERIALS AND METHODS

Request a protocol through *Bio-protocol*.

**Table 1:** Resources data.

Reagent or resource	Source	Identifier
Antibodies		
Anti-p53 [DO-1], mouse monoclonal	Abcam	Catalogue# ab1101, RRID:AB_297667
Anti-p21 Waf1/Cip1 (12D1), rabbit monoclonal	Cell Signaling	Catalogue# 2947, RRID:AB_823586
Anti-GAPDH (D16H11), rabbit monoclonal	Cell Signaling	Catalogue# 5174, RRID:AB_10622025
Anti-β-actin (D6A8), rabbit monoclonal	Cell Signaling	Catalogue #8457, RRID:AB_10950489
Antiphospho-histone H2A.X (Ser-139), rabbit monoclonal	Cell Signaling	Catalogue #9718, RRID:AB_2118009
Anti Muc2	BD Biosciences	Catalogue #555926, RRID:AB_396227
Antirabbit IgG, HRP-linked Antibody	Cell Signaling	Catalogue# 7074, RRID:AB_2099233
Antimouse IgG, HRP-linked Antibody	Cell Signaling	Catalogue# 7076, RRID:AB_330924
Antimouse IgG (H+L), F(ab’)2 Fragment (Alexa Fluor 488 Conjugate)	Cell Signaling	Catalogue# 4408, RRID:AB_ 10694704
Antirabbit IgG (H+L), F(ab’)2 Fragment (Alexa Fluor 555 Conjugate)	Cell Signaling	Catalogue# 4413, RRID:AB_ 10694110
Antimouse IgG (H+L), F(ab’)2 Fragment (Alexa Fluor 647 Conjugate)	Cell Signaling	Catalogue# 4410, RRID:AB_1904023
Alexa Fluor 488 Polyclonal Antibody	Thermo Fisher Scientific	Catalogue# A-11094, RRID:AB_221544
Biological Samples		
Human colon resection samples	This study	
Chemicals, Peptides, and Recombinant Proteins		
NMS-P715	EMD/Millipore	Catalogue# 475949
Nutlin-3a	Sigma Aldrich	Catalogue# N6287
Nocodazole	Sigma Aldrich	Catalogue# M1404
ZM447439	Selleckchem	Catalogue# S1103
Reversine	Cayman Chemical	Catalogue# 10004412
Doxorubicin	Sigma Aldrich	Catalogue# AMBH324A4B72
KU-60019	Selleckchem	Catalogue# S1570
VE-821	Selleckchem	Catalogue# S8007
GSK923295	Selleckchem	Catalogue# S7090
Cytochalasin D	Sigma	Catalogue# C8273
Monastrol	Selleckchem	Catalogue# S8439
MPI-0479605	Selleckchem	Catalogue# S7488
Palbociclib	Selleckchem	Catalogue# S4482
KaryoMAX Colcemid Solution in HBSS	Thermo Fisher Scientific	Catalogue# 15210040
Janelia Fluor HaloTag Ligand 549	Gift from Bin Wu lab	
Critical Commercial Assays		
NEBNext Single Cell Lysis Module	New England Biolabs	Catalogue# E5530S
NEBNext Single Cell/Low Input RNA Library Prep Kit	New England Biolabs	Catalogue# E6420L
NEBNext Multiplex Oligos	New England Biolabs	Catalogue# E6440S
Click-iT EdU Alexa Fluor 488	Invitrogen	Catalogue# C10420
PureLink RNA Mini	Invitrogen	Catalogue# 12183018A
Superscript IV VILO Master Mix	Invitrogen	Catalogue# 11756050
Fast SYBR Green Fast Mix	Applied Biosystems	Catalogue# 4385610
Lenti-X concentrator	Takara Bio	Catalogue# 631232
Recombinant DNA		
pMD2.G	Laboratory of Didier Trono	RRID:Addgene_12259
psPAX2	Laboratory of Didier Trono	RRID:Addgene_12260
pLV-H2B-Neon-T2A-mCherry-CAAX	This study	
pLV-H2B-Neon-T2A-mCherry-53BP1	This study	
pLV-EGFP-Cdt1-T2A-CENPA-Halo	This study	
pLV-H2B-Dendra2	This study	
Tol2 8xSTAR-mScarlet-NLS. PGK-puro	Laboratory of Hugo Snippert	RRID:Addgene 136263
Software and Algorithms		
ImageJ	Schneider *et al.*, 2012	https://imagej.nih.gov/ij/
R, version 3.6.1	R Core Team, 2019	www.R-project.org/
RStudio, version 1.1.463	RStudio Team, 2020	www.rstudio.com/
ggplot2, version 3.2.1	Wickham, 2016	https://ggplot2.tidyverse.org/

### Lead contact

Further information and requests for resources and reagents should be directed to and will be fulfilled by the lead contact, Rong Li (mbihead@nus.edu.sg).

### Patient-derived colon organoid culture

The human colon organoid (colonoid) line used in this study was generated from an ascending colon resection for CRC. All protocols were approved by the Johns Hopkins School of Medicine Institutional Review Board. The mucosa was separated from the underlying tissue and finely minced. To release crypts, EDTA was added to a final concentration of 31.25 mM and the minced tissue was placed on an orbital shaker at 160 rpm for 1 h at 4ºC. Crypts were isolated by differential centrifugation and embedded in Matrigel [Corning]. Colonoids were grown in 24 well plates (or 8-well chambered cover glass [Nunc LabTek II] for live imaging and immunofluorescence experiments) in proliferation media containing 50% Wnt-3a conditioned media, 15% R-spondin-1 conditioned media, 10% Noggin conditioned media, EGF, B27 supplement, A-83-01, SB202190, and primocin. Differentiation media did not contain Wnt-3a, R-spondin-1, or SB202190. Colonoids were passaged every 6–7 d by adding 1 ml 1x TrypLE Express [Life Technologies] directly to each well to dissociate colonoids into single cells. Briefly, colonoids were incubated at 37ºC for 10–15 min with trituration every 5 min until a single cell suspension was generated. Cell number was counted using flow cytometry with propidium iodide staining to exclude dead cells. Single cells were embedded in Matrigel at a density of 600 cells/µl of Matrigel, and 25 µl was added per well in 24-well plates or 15 µl per well in 8-well chambered cover glass. After Matrigel polymerized for 10 min at 37ºC, 500 µl of media was added per well in 24-well plates or 400 µl per well in 8-well chambered cover glass. Ten micrometers of Y-27632 [Sigma] was added to the media for the first 2 d after passaging. For modified colonoids with puromycin resistance, 1 µg/ml puromycin [Life Technologies] was added to media.

### Single-cell RNA sequencing

For single-cell RNA sequencing, wild-type colonoids were passaged as described above. 0.5 µM reversine or 0.05% DMSO was added for 24 h on d 4 after passaging. Reversine and DMSO were then washed out, and colonoids were grown in proliferation media for 24 h. Colonoids were then dissociated into single cells by adding 1 ml 1x TrypLE Express directly to each well. Colonoids were incubated at 37ºC for 20 min with trituration every 5 min until a single cell suspension was generated. Cells were stained with ethidium homodimer to exclude dead cells. Single cells were then sorted directly into 96-well PCR plates containing 5 µl NEBNext cell lysis buffer with murine RNase inhibitor [New England Biolabs] and flash frozen on dry ice. cDNA synthesis, amplification, and library generation were performed according to the NEBNext Single Cell/Low Input RNA Library Prep Kit manufacturer’s protocol [New England Biolabs]. NEBNext multiplex oligos were used as index primers. the Biomek i7 Genomics Workstation. The libraries were sequenced on the NovaSeq 6000 system at PE150 with NovogeneAIT Genomics. Paired reads were then aligned with STAR, and gene expression was quantified with HTSeq to generate a count matrix using Partek Flow software. Sequencing yielded a median of 7742 genes detected and 4,212,297 total counts per cell. Twenty nine cells with < 5000 genes detected or >20% mitochondrial reads were excluded. One cell was excluded following dimensionality reduction. For inference of karyotype from scRNAseq data, we developed a custom pipeline to estimate chromosome-level changes in gene expression patterns which demonstrated reliable performance and higher accuracy compared with competing methods. Briefly, normalized gene counts were summed across all genes on the same chromosome, which were then aggregated across all cells to estimate population median chromosome scores. For each cell in each chromosome, we calculated deviation from this median score and assigned gain (or loss) of chromosome as high (or low) chromosome score. We followed Griffiths *et al.* (2017) in gene expression normalization and chromosome score calculation, followed by permutations of gene-chromosome relationships to estimate the significance of deviation for each (cell, chromosome) pair. Differential expression analysis was completed using DESeq2 (Love *et al.*, 2014). Gene set enrichment analysis was performed using clusterProfiler (Wu *et al.*, 2021). The p53 pathway score was calculated using the “AddModuleScore” function in the Seurat package (Hao *et al.*, 2021). Genes used to calculate p53 pathway score were: *CDKN1A*, *GADD45A*, *PLK2*, *MDM2*, *RPS27L*, *TRIAP1*, and *FAS*.

### Lentivirus

Plasmids used: pMD2.G (gift from Didier Trono (Addgene plasmid # 12259; http://n2t.net/addgene:12259; RRID:Addgene_12259)), psPAX2 (gift from Didier Trono (Addgene plasmid # 12260; http://n2t.net/addgene:12260; RRID:Addgene_12260)), pLV-H2B-Neon-ires-Puro (this study), pLV-H2B-Neon-T2A-mCherry-CAAX-ires-Puro (this study), pLV-H2B-Neon-T2A-mCherry-53BP1-ires-Puro (this study), pLV-H2B-Dendra2-ires-Puro (this study), pLV-eGFP-Cdt1-T2A-CENPA-Halo (this study), Tol2 8xSTAR-mScarlet-NLS. PGK-puro (gift from Hugo Snippert (Addgene plasmid # 136263; http://n2t.net/addgene:136263; RRID:Addgene_136263)). HEK 293FT cells were cotransfected with the lentiviral transfer plasmid, packaging plasmid, and envelope plasmid. Media containing lentivirus was collected 24- and 48 h after transfection. Lentivirus was concentrated using Lenti-X concentrator [Takara Bio, Catalogue# 631232].

### Generation of modified colonoid lines

Lentiviral transduction of colonoids was performed as previously described (Bolhaqueiro *et al.*, 2018). Colonoids were dissociated to single cells and resuspended in 1850 µl colonoid proliferation media, 50 µl concentrated lentivirus, 10 µM Y-27632, and 0.8 µg/ml polybrene. Cells were spinfected for 1 h, 100 rpm, 28ºC. Cells were incubated with virus at 37ºC for 4-6 h then rinsed to remove virus and embedded in Matrigel at a density of 1000 cells/µl Matrigel and grown in media containing 10 µM Y-27632 for the first 2 d after infection. Colonoids were grown for 7 d and then passaged. One microgram per milliliter puromycin was added immediately after the first passage.

### Drug treatments

The following drugs were used in this study: reversine [Cayman Chemical, Catalogue# 10004412], doxorubicin [Sigma Aldrich, Catalogue# AMBH324A4B72], nutlin-3a [Sigma Aldrich, Catalogue# N6287], NMS-P715 [EMD/Millipore, Catalogue# 475949], GSK923295 [Selleckchem, Catalogue# S7090], cytochalasin D [Selleckchem, Catalogue# C8273], ZM-447439 [Selleckchem, Catalogue# S1103], MPI-0479605 [Selleckchem, Catalogue# S7488], monastrol [Selleckchem, Catalogue# S8439], nocodazole [Sigma Aldrich, Catalogue# M1404], KU-60019 [Selleckchem, Catalogue# S1570], VE-821 [Selleckchem, Catalogue# S8007], and palbociclib [Selleckchem, Catalogue# S4482]. Drug washout was performed by rinsing colonoids three times with 1 ml phosphate-buffered saline (PBS). Reversine was added 4 d after colonoid passaging for all experiments using colonoid proliferation media and 3–4 d after passaging for experiments involving colonoid differentiation.

### Metaphase chromosome spreads

Cells were arrested in metaphase by adding 10 µg/ml Karyomax Colcemid [Thermo Fisher Scientific] for 4–6 h. Colonoids were then dissociated using TrypLE Express for 5–10 min at 37ºC. Cells were centrifuged at 300 × g, 5 min. The cell pellet was resuspended in 50 µl PBS then 5 ml 0.56% KCl solution was added for 13 min to promote cell swelling. One hundred and twenty microliters of 3:1 methanol:glacial acetic acid fixative solution was added and cells were pelleted by centrifuging at 300 × g, 5 min. Cells were resuspended in 7 ml 3:1 methanol:glacial acetic acid fixative solution and incubated overnight at 4ºC. Cells were then dropped on glass slides and dried at 65ºC for 20 min. Vectashield mounting media containing DAPI was added to each slide, and a cover glass was placed and sealed. Spreads were imaged on a Nikon TiE-Eclipse epifluorescence microscope (60× oil immersion objective). At least 30 spreads were manually counted per condition. The absolute value of the difference of the chromosome number from the nearest euploid number (46, 92, 184) was calculated for each metaphase spread. This value was then divided by the nearest ploidy (2, 4, 8). The mean of these values for each condition was defined as the “karyotype complexity” and the SD was defined as the “karyotype heterogeneity.”

### Live cell imaging

Colonoids were plated on 8-well chambered cover glass for all live cell imaging experiments. Samples were imaged 4 d after seeding on a Zeiss 780 laser scanning confocal microscope with an environmental chamber maintained at 37ºC with 5% CO_2_ using a 40x water immersion objective (NA 1.1). For imaging mitotic errors, colonoids were imaged every 3–5 min with 15–20 2.0 µm z-slices and cell lines were imaged with 15–20 1.0 µm z-slices. Images were processed using a custom ImageJ macro modified from the ImageJ plugin “Temporal-Color Code.” Mitotic errors were quantified manually. Divisions were classified as “monopolar” when fluorescent chromatin did not separate into two discrete group during anaphase and only a single daughter nucleus formed after mitosis. For imaging 53BP1 foci, colonoids were imaged every 3–5 min with 15–20 1.0 µm z-slices. The number of 53BP1 foci was counted manually.

### Photoconverting and tracking H2B-Dendra2

Colonoids were seeded on 8-well chambered cover glass 4 d before imaging. Fifteen microliters of Matrigel was added to each well at a density of 1200–1500 cells/µl of Matrigel. To bring all colonoids into the same z-plane, the 15 µl of Matrigel was spread in a thin layer across the entire surface of the well. Colonoids were grown in proliferation media supplemented with 10 µM Y-27632 and 1 µg/ml puromycin for the first 2 d. Reversine or vehicle-control was added 1 h before the start of imaging. Total time of reversine treatment was limited to 4 h. Cells were imaged and photoconverted using a Zeiss 780 laser scanning confocal microscope with an environmental chamber maintained at 37ºC with 5% CO_2_ using a 40x water immersion objective (NA 1.1). Cells entering mitosis were manually identified, and 12–16 cells were imaged per round of imaging with two to three rounds of imaging performed per experiment. Mitosis 0 was imaged every 3 min for a total of 24 min with 12 1.0 µM z-slices. The stage position of each mitosis was saved so the same cell could be imaged again later. After each round of Mitosis 0 imaging one daughter cell per observed mitosis was photoconverted from green to red. Photoconversion was performed using 10% 405-nm laser. An approximately 40 × 40 pixel region of interest was scanned 20 times with a pixel dwell time of 3 µsec. Each photoconversion took ∼30 s. After all cells in that round were photoconverted, a 0-h image of the entire colonoid (green and red channels) was collected.

After two to three rounds of Mitosis 0 imaging, each well was rinsed three times with PBS, and 400 µl proliferation media was added to each well. For Mitosis 1 imaging, colonoids were returned to the microscope 12 h after the first round of Mitosis 0 imaging. Colonoids were imaged every 7.5 min for 12 h with 16 2.0 µm z-slices. For 24- and 48-h imaging, colonoids were returned to the microscope at 24- and 48 h after the first round of Mitosis 0 imaging, and each colonoid was imaged then the slide was returned to the microscope. A custom ImageJ macro was used to process the images and movies to maximum intensity Z projections. Division during the Mitosis 1 imaging period and the number of photoconverted progeny in the 24- and 48-h images were quantified manually.

### Western blot

Colonoids were isolated by dissolving the Matrigel at 4ºC in Cell Recovery Solution [Corning] with orbital shaking for 20 min. Cell lines were collected using 0.05% trypsin. Samples were pelleted by centrifugation (300 × g, 5 min), and protein was isolated immediately or the cells were flash-frozen in liquid nitrogen. Cells were resuspended in 50–150 µl of lysis buffer containing 1X RIPA buffer, Roche Complete Mini, Roche PhoSTOP, 5% glycerol, 0.1% SDS. Cells were lysed by vortexing every 5 min for 25 min followed by sonication. Lysates were centrifuged at 16000 × g for 10 min at 4ºC. Supernatants containing protein were collected, and the protein concentration was measured using Bradford dye [BCA, Bio-Rad]. The lysate concentration was normalized, and Bolt LDS Sample Buffer [Invitrogen] and 40 mM dithiothreitol was added. Samples were separated in Bolt 4–12% Bis-Tris precast polyacrylamide gels [Invitrogen]. Proteins were transferred to PVDF membranes [Invitrogen] using the iBlot 2 Dry Blotting system [Invitrogen] Membranes were blocked with Intercept Blocking Buffer [LI-COR] or 5% bovine serum albumin in TBST (chemiluminescent blots). Primary antibodies were diluted in TBST and incubated with membranes overnight at 4ºC with orbital shaking. Membranes were rinsed three times with TBST then secondary antibodies diluted in TBST were added for 1 h at room temperature. Membranes were then rinsed one time with TBST followed by two times with TBS for fluorescent blots and three times with TBST for chemiluminescent blots. Membranes were then immediately imaged for fluorescent blot and imaged after addition of Clarity Western ECL [Bio-Rad] substrate for chemiluminescent blots using a LI-COR Odyssey imager or Invitrogen iBright imager. Relative protein abundances were quantified using ImageJ. Quantification was normalized across biological replicates using sum normalization. To normalize for aneuploidy frequency (Supplemental Figure S1H), relative p53 abundance was divided by the proportion of aneuploid cells in each condition (DMSO: 0.23, 0.25 µM rev: 0.5, 0.5 µM rev: 0.762).

### EdU incorporation

Colonoids were incubated with 10 µM EdU for 12 h (Figure 1D) or 16 h (all other EdU experiments). For flow cytometry, colonoids were dissociated by adding 1 ml TrypLE Express to each well for 20–30 min at 37ºC with trituration every 5 min until a single-cell suspension was achieved. EdU labeling was performed using the manufacturer’s protocol for the Click-iT EdU Alexa Fluor 488 Flow Cytometry Assay Kit [Invitrogen]. EdU incorporation was measured by flow cytometry using the Attune flow analyzer. FCS files were analyzed using FlowJo. For imaging based EdU experiments, colonoids were fixed with 4% paraformaldehyde (PFA), then permeabilized and blocked using the manufacturer’s protocol. Samples were imaged using a Zeiss 780 laser scanning confocal microscope with a 40x water immersion objective (NA 1.1). For quantification, budded colonoids were identified based on a quantification of circularity. Then, the number of EdU positive buds was manually quantified in budded colonoids.

### Immunofluorescence

Colonoids were plated on 8-well chambered cover glass for immunofluorescence experiments. Media was removed and colonoids were rinsed one time with PBS. Then, colonoids were fixed in Matrigel with 4% PFA for 10 min at 37ºC. The fixed colonoids were rinsed three times with PBS for 10 min. Colonoids were permeabilized with 0.5% Triton X-100 in PBS and blocked with 10% FBS and 0.1% Triton X-100 in PBS. Primary antibodies were diluted in 2% FBS and 0.1% Triton X-100 in PBS and added to samples overnight at 4ºC. Colonoids were then washed three times with PBS. Secondary antibodies diluted in 2% FBS and 0.1% Triton X-100 were added to samples for 2 h at room temperature. Samples were rinsed one time with PBS, then 1 µg/ml DAPI was added for 10 min at room temperature, and samples were rinsed one time with PBS. Samples were imaged using a Zeiss 780 laser scanning confocal microscope with a 40x water immersion objective (NA 1.1). For the Muc2 immunofluorescence experiment in Figure 4, colonoids were plated on ibidi 8-well µ-slides, DNA was stained via 1 µg/ml Hoechst 33342, and samples were imaged using a Zeiss LSM980 Airyscan laser scanning confocal microscope in Multiplex SR-4Y mode with a 40x water immersion objective (NA 1.2). Muc2 positive and EdU-positive cells were manually counted using a single Z slice.

### CENPA foci counting

Cdt1-eGFP-T2A-CENPA-Halo colonoids were plated as described in the immunofluorescence section. Colonoids were then treated with drugs for 24 h followed by 24 h in drug-free proliferation media. Then, 10 nM Janelia Fluor HaloTag Ligand 549 [gift from Bin Wu lab] in proliferation media was added to the colonoids for 30 min followed by three 10-min washes with proliferation media containing no fluorophore. Colonoids were then fixed and stained with anti-p53 antibody, as described in the immunofluorescence section above. An AlexaFluor 488 secondary antibody and DAPI were used. Samples were then imaged using a Zeiss LSM 880 microscope in SR mode with a 63x oil immersion objective.

The process of chromosome counting consists of two main parts: 1.) thresholding and segmenting; 2.) Counting. First, cell nuclei are identified and segmented based on the eGFP-Cdt1 signal intensity. Subsequently, within each nucleus, the centromeres are recognized and segmented using the CENPA-Halo signal intensity. Otsu’s method (Sezgin and Sankur, 2004) is employed for automatic thresholding in both image segmentation processes to differentiate the foreground signal from the background. In the counting process, the CENPA-Halo signal is initially binarized after thresholding. For the processed signal, we find and count the connected components in the binary image, which represents the chromosome clusters. To determine the chromosome count in each cluster, we adopt a signal intensity-based approach. For each cluster, the total signal intensity (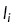) is calculated. The median intensity (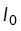) is approximated as the mean intensity of a single centromere, assuming most centromeres are separated. The total chromosome number (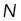) is then calculated as: 
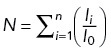
, where 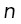 is the number of chromosome clusters. The resulting count for each cluster is rounded to the nearest integer. Given the inherent variation in individual chromosome intensity, chromosome clusters with higher or lower intensity levels may introduce larger errors in the counting process. To address this issue, we adopt a mitigation strategy by utilizing the number of local intensity maxima to estimate the chromosome count for the three clusters with the highest intensity. Additionally, we apply this approach to clusters whose intensity is lower than the mean intensity (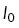). Mean p53 intensity was calculated using the nuclear segmentation. In Figure 2K, cells with ≤47 CENPA foci are considered euploid, 48 or 49 foci are simple aneuploid, and ≥50 foci are complex aneuploid.

### Organoid formation assay

Colonoids were dissociated by adding 1 ml TrypLE Express to each well for 20–30 min at 37ºC with trituration every 5 min until a single-cell suspension was achieved. Cell number was counted using flow cytometry with propidium iodide staining to exclude dead cells. Cells were embedded in Matrigel at a density of 600 cells/µl of Matrigel, and 15 µl was plated per well on 8-well chambered cover glass. Colonoids were grown in colonoid proliferation media supplemented with 10 µM Y-27632 for the first 2 d. For fixed imaging, colonoids were fixed with 4% PFA for 10 min at 37˚C. Colonoids were rinsed three times with PBS, then permeabilized for 1 h at room temperature with 0.5% Triton X-100 in PBS. Samples were rinsed one time with PBS then 1 µg/ml DAPI was added for 10 min at room temperature, and samples were rinsed one time with PBS. For tracking of colonoid growth in live cells, colonoids were imaged every 2 d and positions were saved to image the same colonoids each day. Samples were imaged using a Zeiss 780 laser scanning confocal microscope with a 10x objective for fixed cells and 20x objective for live cells.

### RT-qPCR

Colonoids were collected by adding 500 µl TRIzol reagent [Invitrogen] directly to each well of colonoids in Matrigel. This solution was flash frozen in liquid nitrogen and stored at –80ºC until RNA extraction. RNA was extracted using the manufacturer’s protocol for the PureLink RNA mini kit [Invitrogen]. RNA concentration was measured using a NanoDrop spectrophotometer. RNA was converted to cDNA using the Superscript IV VILO Master Mix [Invitrogen]. RT-qPCR reactions were prepared using the Fast SYBR Green Fast Mix [Applied Biosystems]. Quantification was performed using the ΔΔC_t_ method.

### *ASCL2* reporter

Colonoids were transduced with Tol2 8xSTAR-mScarletI-NLS. PGK-puro and selected with 1 µg/ml puromycin. Colonoids expressing the reporter were treated with 0.05% DMSO or 0.25 µM reversine in proliferation media for 24 h. Drugs were washed out 3x with PBS, and differentiation media was added for 96 h with daily media changes. After 96 h, colonoids were dissociated into single cells with 1x TrypLE Express and directly analyzed via flow cytometry. Wild-type colonoids were used to establish a cutoff for reporter positivity.

### General Statistics

The number of biological replicates, the number of cells analyzed per biological replicate, and the statistical tests performed are indicated in the figure legends. A biological replicate was defined as a unique culture of colonoids. Statistical tests were performed, and figures were generated using R-Studio.

## Supplementary Material



## Abbreviations used:

HiRev: 0.5 µM reversine
LoRev: 0.25 µM reversine
