# Supplementary material for: Simple aneuploidy evades p53 surveillance and promotes niche factor-independent growth in human intestinal organoids
Source: Mol Biol Cell. 2024 Jul 22;35(8):br15. doi: 10.1091/mbc.E24-04-0166 (PMC11321050; doi:10.1091/mbc.E24-04-0166)
Supplement: Supplementary file 2 [file mbc-35-br15-s001.pdf]

Supplemental Materials

Molecular Biology of the Cell

Johnson *et al*.

Supplemental information titles and legends

Supplemental Table 1. Simple and complex aneuploidy gene expression

Table shows expression comparison of all genes from scRNA-seq data. Simple aneuploid cells (1-3 chromosome changes) are compared with euploid cells and complex aneuploid cells (≥4 chromosome changes) are compared with euploid cells. Both comparisons are shown in this table. Cutoffs for differential expression were $\log_2$(fold change) > 1 or < -1 and FDR ≤ 0.5. Genes with positive $\log_2$(fold change) are more highly expressed in aneuploid cells. Table is the output from DESeq2.

Supplemental Video 1. Representative time-lapse H2B-Dendra2 tracking movie demonstrating division after chromosome mis-segregation, related to Figure 3. Mitosis 0 time interval: 3 minutes 18 seconds, Mitosis 1 time interval: 5 minutes. Mitosis 1 imaging started 12 hours after mitosis 0. Non-photoconverted H2B-Dendra2 shown in green. Photoconverted H2B-Dendra2 shown in magenta. Mitosis 1 images were zoomed in to the photoconverted cell at the time of imaging.

Supplemental Figure 1. Generating and inducing aneuploidy in colonoids

(A) Representative H&E stained tissue section from the colon resection sample used to generate the colonoid line used in this study.

(B) (left) Immunoblot of the human colonoid line used in this study treated with increasing concentrations of Nutlin-3a for 24 hours. GAPDH is a loading control. (right) Bar plot showing p21 abundance relative to GAPDH. n = 1 biological replicate.

(C) Images of cultures of the human colonoid line used in this study one week after passaging in the presence of increasing concentrations of Nutlin-3a.

(D) (top) Percentage of diploid (46 chromosomes), simple aneuploid (1-3 chromosome changes), complex aneuploid ($\geq$ 4 chromosome changes), or tetraploid (92 chromosomes) metaphase spreads following treatment with aneuploidy-inducing drugs for 24 hours followed by 24 hours in drug-free proliferation media. Number of metaphase spreads shown above bar. (bottom) Karyotype complexity and heterogeneity of conditions on left. ZM = ZM447439, CEi = CENPE inhibitor (GSK923295), CytoD = cytochalasin D, NMS and N = NMS-P715, Rev = reversine, Noc = nocodazole

(E) Cumulative frequency plot of the nuclear envelope breakdown to anaphase onset time in human colon colonoids treated with 0.05% DMSO, 0.25 or 0.5 µM reversine. The time from NEBD-anaphase onset was calculated from live cell imaging of H2B-Neon or H2B-Neon-T2A-mCherry-CAAX colonoids. Combined data from $\geq$ 3 experiments.

(F) (left) Representative images of the mitotic errors. (right) Quantification of the frequency of mitotic errors observed in live cell images in human colonoids. Each dot represents the frequency of error in one experiment. Bars represent the mean of all experiments. $n \geq 2$ biological replicates and $n \geq 40$ divisions per condition. Fisher's exact test. *$p < 0.05$, **$p < 0.01$. lc = lagging chromosome, cb = chromatin bridge, mono = monopolar

(G) Replicates 2 and 3 of immunoblots of human colonoids treated with DMSO (vehicle-control) or reversine for 24 hours followed by 0, 24, 48, 72, or 96 hours in drug-free proliferation media.

Replicate 1 is shown in Figure 1B Quantification of 24 h drug-free timepoint is shown in Figure 1C. GAPDH is a loading control. Membrane was cut prior to antibody staining and cut edges are visible in the presented images. Two membranes were needed due to the number of samples in this experiment. Separate membranes denoted by vertical black line.

(H) p53 quantified by Western blot and normalized by aneuploidy frequency (Figure 1C, see Methods) in colonoids treated for 24 hours followed by 24 hours in drug-free proliferation media. n = 3 biological replicates. Error bars represent standard deviation. Abundances compared using ANOVA and post-hoc Tukey test. Tukey p values are shown.

(I) Immunoblot of human colonoids treated with NMS-P715 or reversine for 24 hours followed by 24 hours in drug-free media. Nutlin-3a and doxorubicin were positive controls. GAPDH is a loading control. n = 1 biological replicate. Membrane was cut prior to antibody staining and cut edges are visible in the presented images.

Supplemental Figure 2. Single cell RNA sequencing clustering and pathway analysis

(A) UMAP of scRNA-seq data. Cells with 1-3 aneuploid chromosomes are considered simple aneuploid (blue) and cells with ≥ 4 aneuploid chromosomes are considered complex aneuploid (red).

(B) UMAP of scRNA-seq data. Colors represent cell cycle stage.

(C) Gene set enrichment analysis of KEGG pathways utilizing all genes for complex aneuploid cells in scRNA-seq dataset.

(D) Gene set enrichment analysis of KEGG pathways utilizing all genes for simple aneuploid cells in scRNA-seq dataset.

(E) p53 pathway score from scRNA-seq data comparing cells with loss, gain, or no change in chromosome 17. p53 scores compared using ANOVA and post-hoc Tukey test. Tukey p values are shown.

(F) Karyotype distributions of chromosome numbers counted using CENPA-Halo foci. n = 3 biological replicates. n = 29 cells for DMSO, 22 cells for 0.25 µM reversine and 87 cells for 0.5 µM reversine. Blue dashed line at euploid and tetraploid chromosome numbers.

(G) Mean p53 fluorescence intensity in CENPA-Halo-eGFP-Cdt1 colonoids treated with DMSO, reversine (rev), or 100 nM doxorubicin (dox). Dots represent individual nuclei, bar represents mean. n = 3 biological replicates (n = 2 replicates dox).

Supplemental Figure 3. p53 activation in aneuploid populations is not driven by DNA damage

(A) (top) Representative images of colonoids immunostained for p53 and pH2AX following 24 h drug treatment and 24 h in drug free media. (bottom) Quantification of p53 positive, pH2AX positive, and p53-pH2AX co-positive cells.

(B) (top) Representative images from H2B-Neon-T2A-mCherry-53BP1 movies. (bottom) Quantification of 53BP1 foci following mitosis. Line represents the mean number of 53BP1 foci per cell and ribbon represents standard deviation. n = 3 biological replicates, n ≥ 50 cells for DMSO and reversine and n = 14 cells for doxorubicin. Scale bar = 5 µm.

(C) (left) Representative images of colonoids immunostained for p53 and pH2AX following 4 h drug treatment and 16 h in drug free media. (right) Quantification of mean pH2AX IF intensity in p53 positive and p53 negative nuclei determined by IF in colonoids treated with DMSO, LoRev, HiRev, or 100 nM doxorubicin for 4 hours followed by 16 hours in drug-free media. n = 3 biological replicates (n = 2 for doxorubicin). Dots with color represent mean of each biological replicate. Unfilled dots represent individual nuclei. p values calculated using Welch’s two sample t test.

(D) (top) p53 and p21 immunoblot with or without ATM (2 µM KU-60019) and ATR (2 µM VE-821) inhibition for 24 (after reversine treatment) or 48 (during and after reversine treatment) hours. (bottom) Quantification of p53 and p21 abundance n = 3 biological replicates. p values calculated by Welch’s two sample t test.

(E) Quantification of EdU positivity by flow cytometry with or without ATM (2 µM KU-60019) and ATR (2 µM VE-821) inhibition for 24 or 48 hours. p values calculated by Welch’s two sample t test.

Supplemental Figure 4. Tracking daughter cells following chromosome mis-segregation

(A) Division frequency of photoconverted cells with or without micronuclei. Total number of cells tracked is shown above each bar. p value calculated using Fisher's exact test.

(B) (left) Quantification of the number of 53BP1 foci per nucleus per colonoid in H2B-Neon-T2A-mCherry-53BP1 colonoids after 0 and 4 hours of imaging. Foci number compared between timepoints using Welch's two sample t test. ≥8 colonoids per condition. (right) Representative image of chromatin bridge without 53BP1 foci in HiRev H2B-Neon-T2A-mCherry-53BP1 colonoid. Scale bar = 10 µm.

(C) Cumulative frequency plot of the time from Mitosis 0 to Mitosis 1 in photoconverted H2B-Dendra2 cells.

(D) Quantification of cell death in photoconverted cells from Figure 3E. The color indicates if cell death was first observed in the 24 hour or 48 hour image. Number of observations shown above each bar. p value calculated using Fisher's exact test comparing each bar to control.

(E) (left) Representative images of a chromatin bridge in Mitosis 0 that led to a binucleate, multipolar division in Mitosis 1. White arrowhead shows which daughter cell was photoconverted. Scale bar = 10 µm, inset scale bar = 5 µm. (right) Quantification of the frequency of bipolar and multipolar divisions in tetraploid cells that divided in Mitosis 1. Number of observations shown above bar.

n = normal mitosis, lc = lagging chromosome, cb = chromatin bridge, mn = micronuclei, mono = monopolar, binuc = binucleate

Supplemental Figure 5. Aneuploidy impairs intestinal stem cell differentiation

(A) Expression of stem cell markers measured by qPCR over time following treatment with vehicle-control (0.05% DMSO) or 0.25 µM reversine. DMSO and 0.25 µM reversine were washed out at time 0 hours and colonoids were grown in proliferation media for the remainder of the experiment. Points represent the mean of 3 biological replicates. Error bars represent standard deviation. p values were calculated using Welch's two sample t test.

(B) Expression of stem cell markers measured by qPCR over time following treatment with vehicle-control or 0.25 µM reversine. DMSO and 0.25 µM reversine were washed out at time 0 hours and colonoids were grown in differentiation media for the remainder of the experiment. Points represent the mean of 3 biological replicates. Error bars represent standard deviation. p values were calculated using Welch's two sample t test.

(C) (left) Quantification of circularity in colonoids treated with vehicle-control or 0.25 µM reversine then grown in drug-free proliferation or differentiation media with 96 hours. n = 3 biological replicates (right) Quantification of the frequency of colonoids that were budded after 96 hours in drug-free proliferation or differentiation media. colonoids were considered budded if their circularity was < 0.925. n = 3 biological replicates. p values were calculated using Fisher's exact test.

(D) Quantification of H2B-Dendra2 mean colonoid area over time. Drugs were added on Day -1 and drugs were washed out and differentiation media was added on Day 0. Line represents mean of 2 technical replicates.

(E) Quantification of circularity in colonoids treated with drugs shown for 24 hours then grown in differentiation media with 96 hours. n = 1 biological replicates. Each dot represents one colonoid. Line denotes mean circularity.

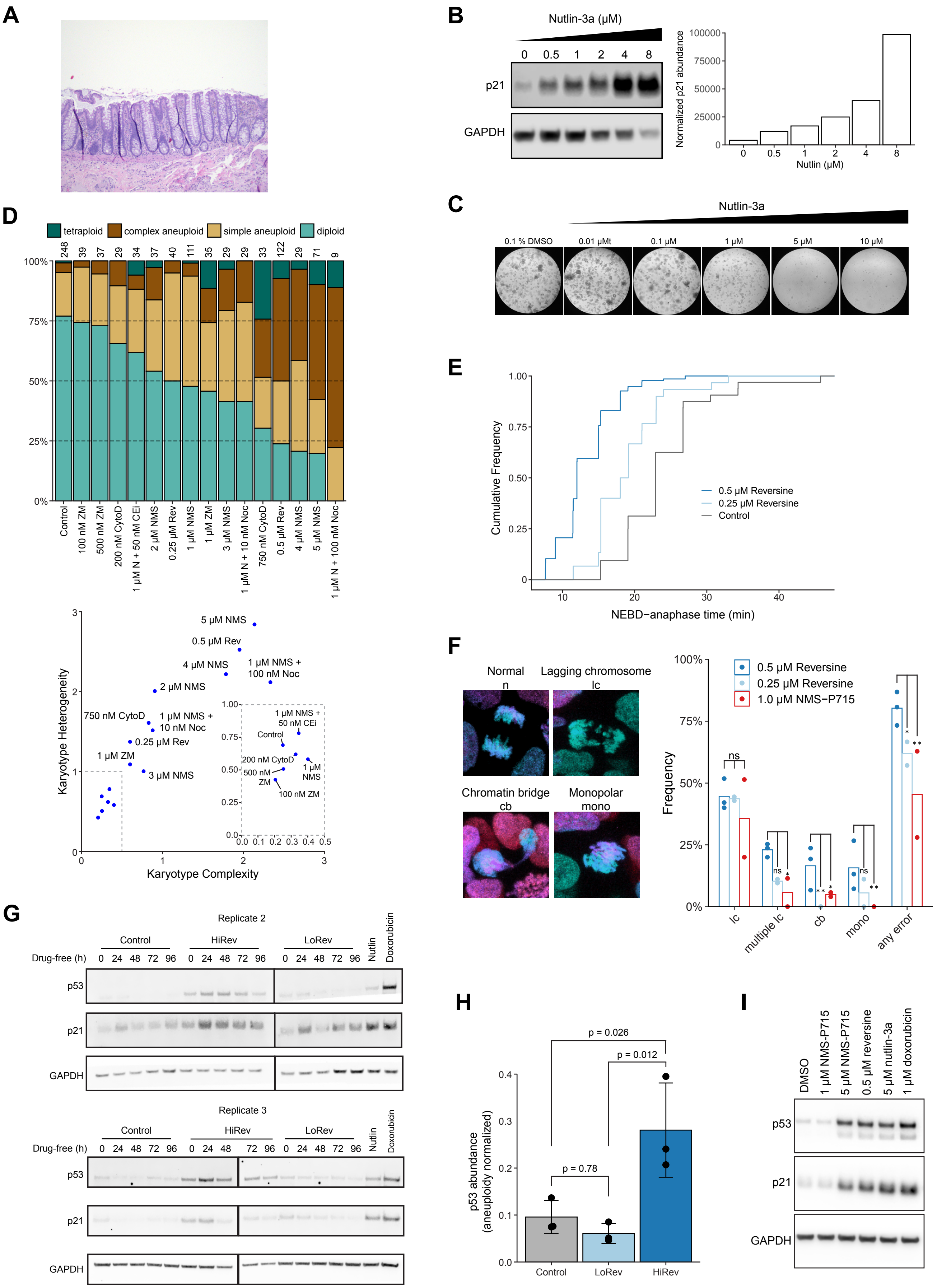

A
B
Nutlin-3a (μM)
0 0.5 1 2 4 8
p21
GAPDH
Normalized p21 abundance
Nutlin (μM)
C
Nutlin-3a
0.1 % DMSO
0.01 μMt
0.1 μM
1 μM
5 μM
10 μM
D
tetraploid
complex aneuploid
simple aneuploid
diploid
Control
100 nM ZM
500 nM ZM
200 nM CytoD
1 μM N + 50 nM CEi
2 μM NMS
0.25 μM Rev
1 μM NMS
1 μM ZM
3 μM NMS
1 μM N + 10 nM Noc
750 nM CytoD
0.5 μM Rev
4 μM NMS
5 μM NMS
1 μM N + 100 nM Noc
Karyotype Heterogeneity
Karyotype Complexity
E
Cumulative Frequency
0.5 μM Reversine
0.25 μM Reversine
Control
NEBD−anaphase time (min)
F
Normal
n
Lagging chromosome
lc
Chromatin bridge
cb
Monopolar
mono
Frequency
0.5 μM Reversine
0.25 μM Reversine
1.0 μM NMS−P715
lc
multiple lc
cb
mono
any error
G
Replicate 2
Replicate 3
Control
HiRev
LoRev
Nutlin
Doxorubicin
Drug-free (h)
p53
p21
GAPDH
H
p = 0.026
p = 0.012
p = 0.78
p53 abundance (aneuploidy normalized)
Control
LoRev
HiRev
I
DMSO
1 μM NMS-P715
5 μM NMS-P715
0.5 μM reversine
5 μM nutlin-3a
1 μM doxorubicin
p53
p21
GAPDH

Supplemental FIgure 1. Generating and inducing aneuploidy in colonoids

(A) Representative H&E stained tissue section from the colon resection sample used to generate the colonoid line used in this study.

(B) (left) Immunoblot of the human colonoid line used in this study treated with increasing concentrations of Nutlin-3a for 24 hours. GAPDH is a loading control. (right) Bar plot showing p21 abundance relative to GAPDH. n = 1 biological replicate.

(C) Images of cultures of the human colonoid line used in this study one week after passaging in the presence of increasing concentrations of Nutlin-3a.

(D) (top) Percentage of diploid (46 chromosomes), simple aneuploid (1-3 chromosome changes), complex aneuploid (≥ 4 chromosome changes), or tetraploid (92 chromosomes) metaphase spreads following treatment with aneuploidy-inducing drugs for 24 hours followed by 24 hours in drug-free proliferation media. Number of metaphase spreads shown above bar. (bottom) Karyotype complexity and heterogeneity of conditions on left. ZM = ZM447439, CEi = CENPE inhibitor (GSK923295), CytoD = cytochalasin D, NMS and N = NMS-P715, Rev = reversine, Noc = nocodazole

(E) Cumulative frequency plot of the nuclear envelope breakdown to anaphase onset time in human colon colonoids treated with 0.05% DMSO, 0.25 or 0.5 μM reversine. The time from NEBD-anaphase onset was calculated from live cell imaging of H2B-Neon or H2B-Neon-T2A-mCherry-CAAX colonoids. Combined data from ≥ 3 experiments.

(F) (left) Representative images of the mitotic errors. (right) Quantification of the frequency of mitotic errors observed in live cell images in human colonoids. Each dot represents the frequency of error in one experiment. Bars represent the mean of all experiments. n ≥ 2 biological replicates and n ≥ 40 divisions per condition. Fisher's exact test. $*p < 0.05$, $**p < 0.01$.

lc = lagging chromosome, cb = chromatin bridge, mono = monopolar

(G) Replicates 2 and 3 of immunoblots of human colonoids treated with DMSO (vehicle-control) or reversine for 24 hours followed by 0, 24, 48, 72, or 96 hours in drug-free proliferation media. Replicate 1 is shown in Figure 1B Quantification of 24 h drug-free timepoint is shown in Figure 1C. GAPDH is a loading control. Membrane was cut prior to antibody staining and cut edges are visible in the presented images. Two membranes were needed due to the number of samples in this experiment. Separate membranes denoted by vertical black line.

(H) p53 quantified by Western blot and normalized by aneuploidy frequency (Figure 1C, see Methods) in colonoids treated for 24 hours followed by 24 hours in drug-free proliferation media. n = 3 biological replicates. Error bars represent standard deviation. Abundances compared using ANOVA and post-hoc Tukey test. Tukey p values are shown.

(I) Immunoblot of human colonoids treated with NMS-P715 or reversine for 24 hours followed by 24 hours in drug-free media. Nutlin-3a and doxorubicin were positive controls. GAPDH is a loading control. n = 1 biological replicate. Membrane was cut prior to antibody staining and cut edges are visible in the presented images.

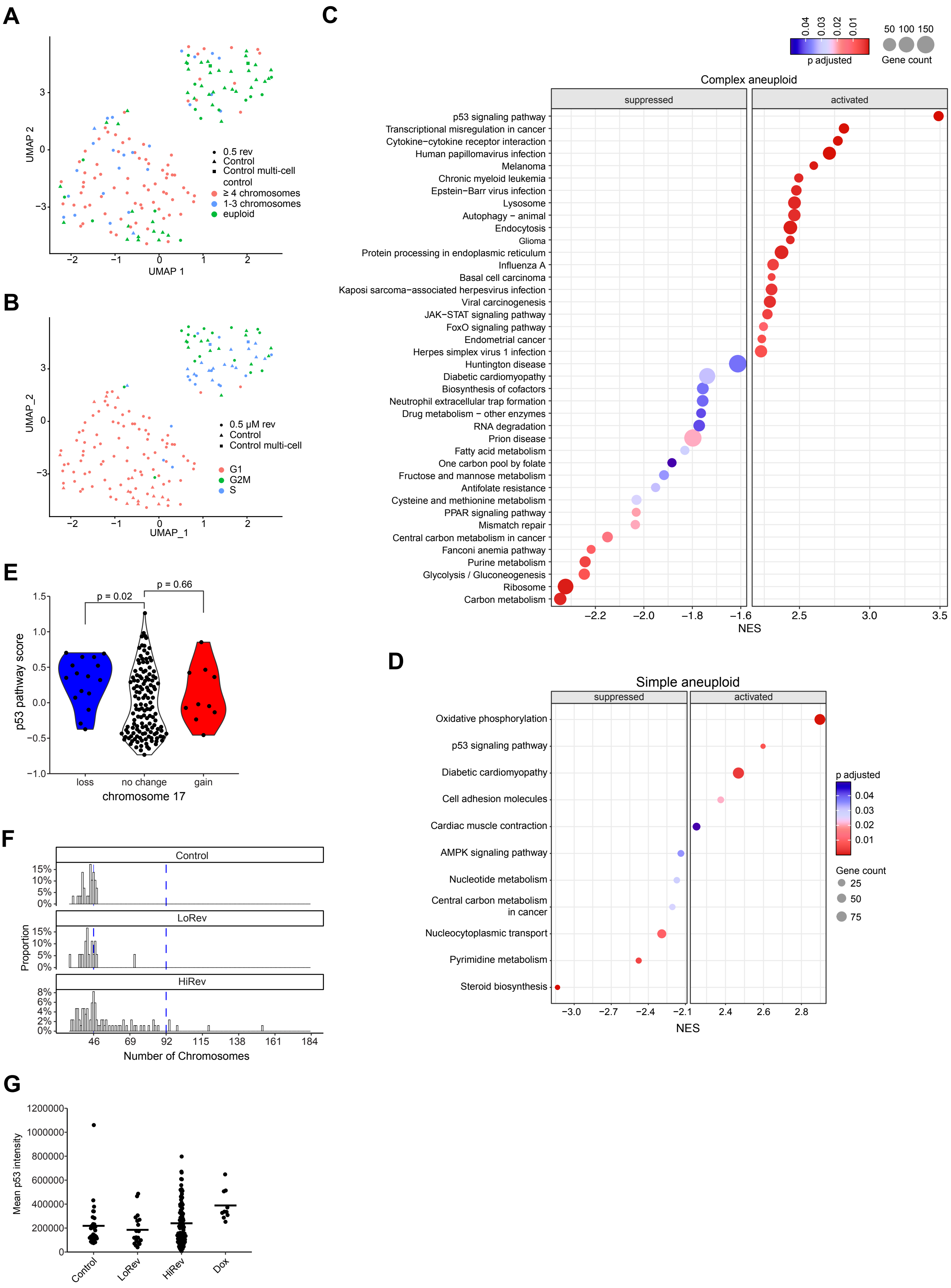

A
UMAP 2
UMAP 1
0.5 rev
Control
Control multi-cell control
≥ 4 chromosomes
1-3 chromosomes
euploid
B
UMAP_2
UMAP_1
0.5 μM rev
Control
Control multi-cell
G1
G2M
S
C
p adjusted
Gene count
Complex aneuploid
suppressed
activated
p53 signaling pathway
Transcriptional misregulation in cancer
Cytokine−cytokine receptor interaction
Human papillomavirus infection
Melanoma
Chronic myeloid leukemia
Epstein−Barr virus infection
Lysosome
Autophagy − animal
Endocytosis
Glioma
Protein processing in endoplasmic reticulum
Influenza A
Basal cell carcinoma
Kaposi sarcoma−associated herpesvirus infection
Viral carcinogenesis
JAK−STAT signaling pathway
FoxO signaling pathway
Endometrial cancer
Herpes simplex virus 1 infection
Huntington disease
Diabetic cardiomyopathy
Biosynthesis of cofactors
Neutrophil extracellular trap formation
Drug metabolism − other enzymes
RNA degradation
Prion disease
Fatty acid metabolism
One carbon pool by folate
Fructose and mannose metabolism
Antifolate resistance
Cysteine and methionine metabolism
PPAR signaling pathway
Mismatch repair
Central carbon metabolism in cancer
Fanconi anemia pathway
Purine metabolism
Glycolysis / Gluconeogenesis
Ribosome
Carbon metabolism
NES
D
Simple aneuploid
suppressed
activated
Oxidative phosphorylation
p53 signaling pathway
Diabetic cardiomyopathy
Cell adhesion molecules
Cardiac muscle contraction
AMPK signaling pathway
Nucleotide metabolism
Central carbon metabolism in cancer
Nucleocytoplasmic transport
Pyrimidine metabolism
Steroid biosynthesis
NES
p adjusted
Gene count
E
p = 0.02
p = 0.66
p53 pathway score
loss
no change
gain
chromosome 17
F
Control
LoRev
HiRev
Proportion
Number of Chromosomes
G
Mean p53 intensity
Control
LoRev
HiRev
Dox

Supplemental Figure 2. Single cell RNA sequencing clustering and pathway analysis
(A) UMAP of scRNA-seq data. Cells with 1-3 aneuploid chromosomes are considered simple aneuploid (blue) and cells with ≥ 4 aneuploid chromosomes are considered complex aneuploid (red).
(B) UMAP of scRNA-seq data. Colors represent cell cycle stage.
(C) Gene set enrichment analysis of KEGG pathways utilizing all genes for complex aneuploid cells in scRNA-seq dataset.
(D) Gene set enrichment analysis of KEGG pathways utilizing all genes for simple aneuploid cells in scRNA-seq dataset.
(E) p53 pathway score from scRNA-seq data comparing cells with loss, gain, or no change in chromosome 17. p53 scores compared using ANOVA and post-hoc Tukey test. Tukey p values are shown.
(F) Karyotype distributions of chromosome numbers counted using CENPA-Halo foci. n = 3 biological replicates. n = 29 cells for DMSO, 22 cells for 0.25 μM reversine and 87 cells for 0.5 μM reversine. Blue dashed line at euploid and tetraploid chromosome numbers.
(G) Mean p53 fluorescence intensity in CENPA-Halo-eGFP-Cdt1 colonoids treated with DMSO, reversine (rev), or 100 nM doxorubicin (dox). Dots represent individual nuclei, bar represents mean. n = 3 biological replicates (n = 2 replicates dox).

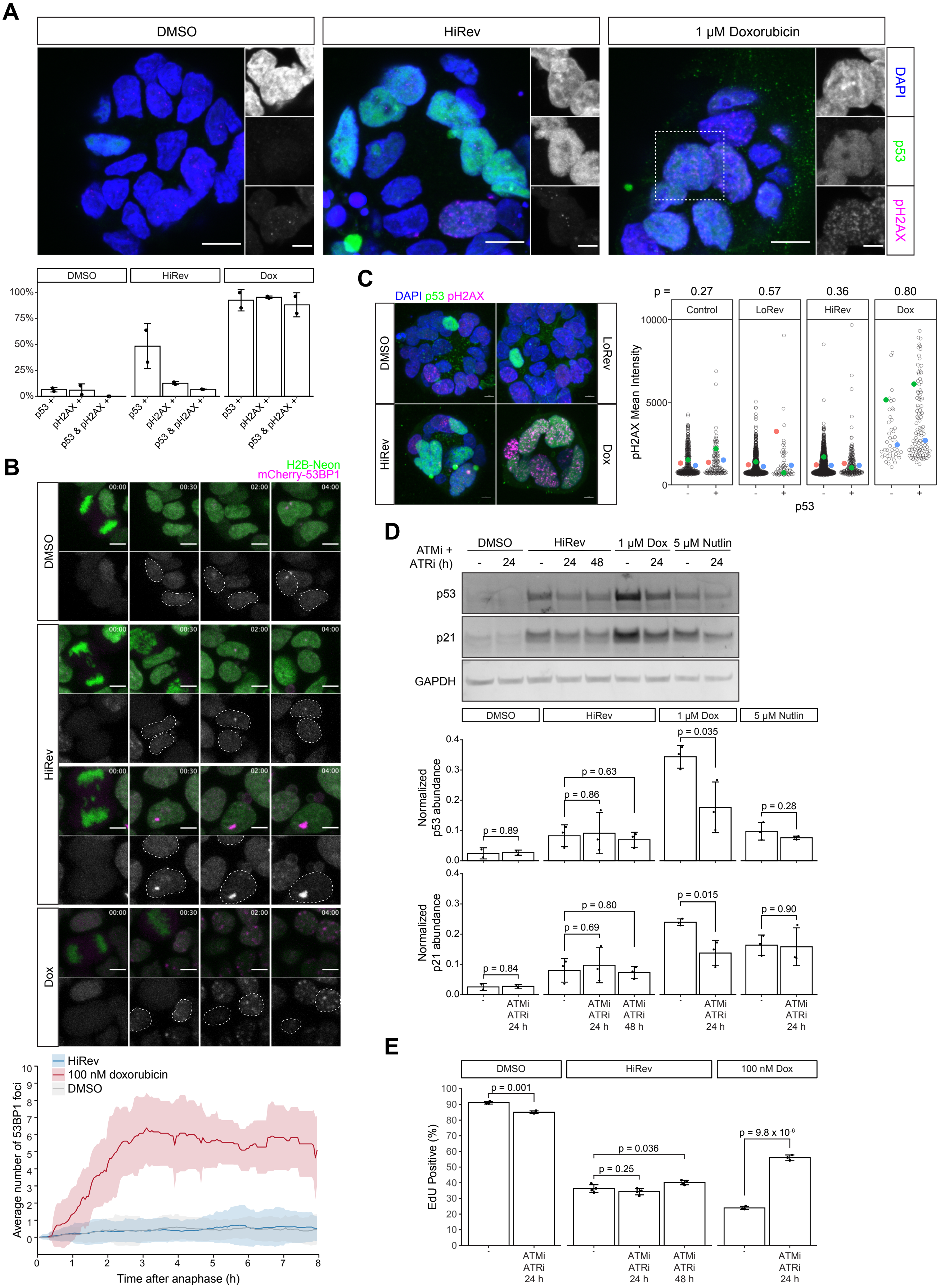
A
DMSO
HiRev
1 μM Doxorubicin
DAPI
p53
pH2AX
DMSO
HiRev
Dox
100%
75%
50%
25%
0%
p53 +
pH2AX +
p53 & pH2AX +
B
H2B-Neon
mCherry-53BP1
DMSO
HiRev
Dox
00:00
00:30
02:00
04:00
HiRev
100 nM doxorubicin
DMSO
Average number of 53BP1 foci
Time after anaphase (h)
C
DAPI p53 pH2AX
DMSO
LoRev
HiRev
Dox
p = 0.27 0.57 0.36 0.80
Control
LoRev
HiRev
Dox
pH2AX Mean Intensity
p53
D
ATMi + ATRi (h)
DMSO
HiRev
1 μM Dox
5 μM Nutlin
p53
p21
GAPDH
Normalized p53 abundance
p = 0.89
p = 0.86
p = 0.63
p = 0.035
p = 0.28
Normalized p21 abundance
p = 0.84
p = 0.69
p = 0.80
p = 0.015
p = 0.90
ATMi ATRi 24 h
ATMi ATRi 48 h
E
DMSO
HiRev
100 nM Dox
EdU Positive (%)
p = 0.001
p = 0.25
p = 0.036
p = 9.8 x 10-6
ATMi ATRi 24 h
ATMi ATRi 48 h

Supplemental Figure 3. p53 activation in aneuploid populations is not driven by DNA damage
(A) (top) Representative images of colonoids immunostained for p53 and pH2AX following 24 h drug treatment and 24 h in drug free media. (bottom) Quantification of p53 positive, pH2AX positive, and p53-pH2AX co-positive cells.
(B) (top) Representative images from H2B-Neon-T2A-mCherry-53BP1 movies. (bottom) Quantification of 53BP1 foci following mitosis. Line represents the mean number of 53BP1 foci per cell and ribbon represents standard deviation. n = 3 biological replicates, n ≥ 50 cells for DMSO and reversine and n = 14 cells for doxorubicin. Scale bar = 5 μm.
(C) (left) Representative images of colonoids immunostained for p53 and pH2AX following 4 h drug treatment and 16 h in drug free media. (right) Quantification of mean pH2AX IF intensity in p53 positive and p53 negative nuclei determined by IF in colonoids treated with DMSO, LoRev, HiRev, or 100 nM doxorubicin for 4 hours followed by 16 hours in drug-free media. n = 3 biological replicates (n = 2 for doxorubicin). Dots with color represent mean of each biological replicate. Unfilled dots represent individual nuclei. p values calculated using Welch's two sample t test.
(D) (top) p53 and p21 immunoblot with or without ATM (2 μM KU-60019) and ATR (2 μM VE-821) inhibition for 24 (after reversine treatment) or 48 (during and after reversine treatment) hours. (bottom) Quantification of p53 and p21 abundance n = 3 biological replicates. p values calculated by Welch's two sample t test.
(E) Quantification of EdU positivity by flow cytometry with or without ATM (2 μM KU-60019) and ATR (2 μM VE-821) inhibition for 24 or 48 hours. p values calculated by Welch's two sample t test.

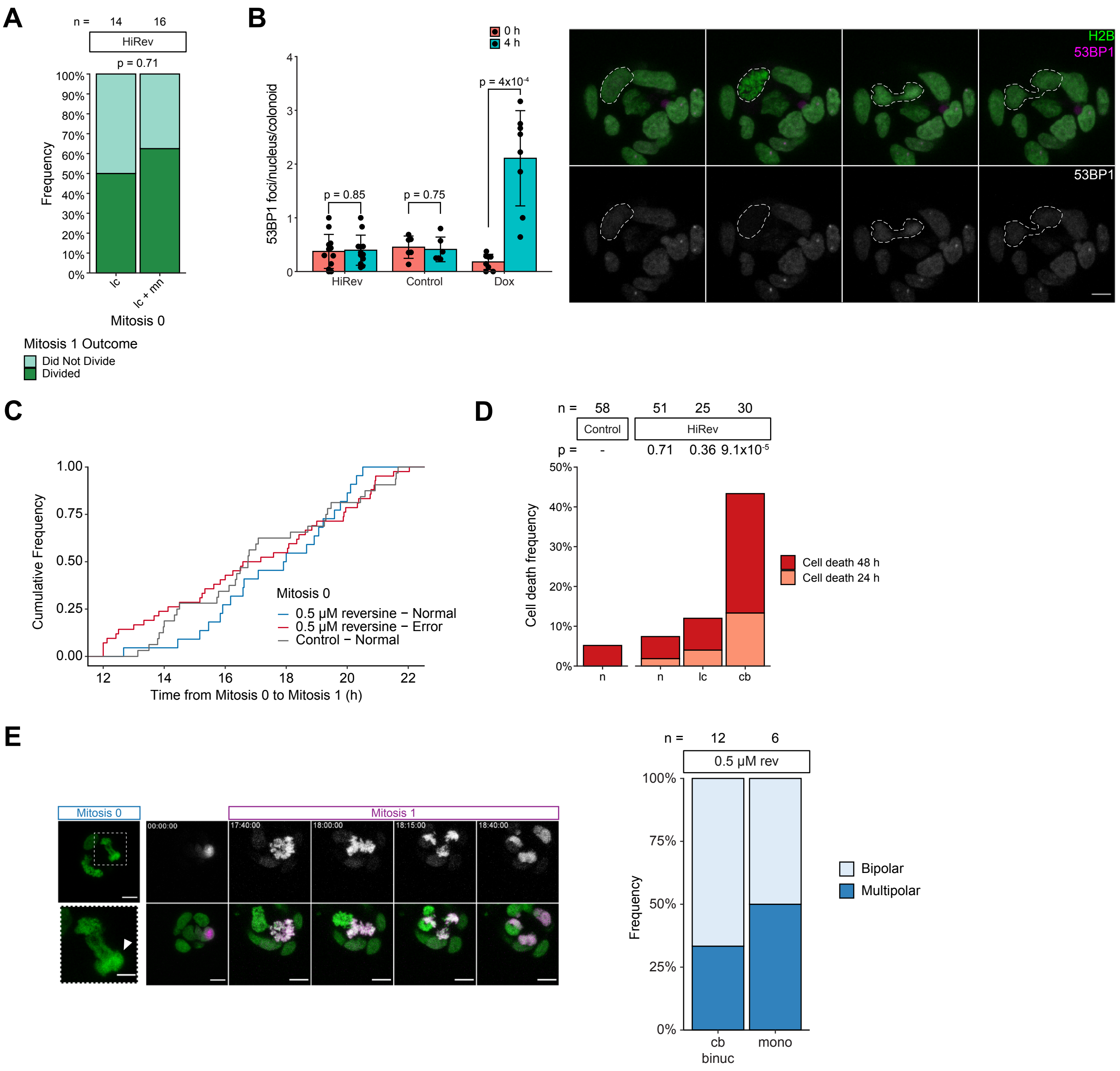

Supplemental Figure 4. Tracking daughter cells following chromosome mis-segregation

(A) Division frequency of photoconverted cells with or without micronuclei. Total number of cells tracked is shown above each bar. p value calculated using Fisher's exact test.

(B) (left) Quantification of the number of 53BP1 foci per nucleus per colonoid in H2B-Neon-T2A-mCherry-53BP1 colonoids after 0 and 4 hours of imaging. Foci number compared between timepoints using Welch's two sample t test. ≥8 colonoids per condition. (right) Representative image of chromatin bridge without 53BP1 foci in HiRev H2B-Neon-T2A-mCherry-53BP1 colonoid. Scale bar = 10 μm.

(C) Cumulative frequency plot of the time from Mitosis 0 to Mitosis 1 in photoconverted H2B-Dendra2 cells.

(D) Quantification of cell death in photoconverted cells from Figure 3E. The color indicates if cell death was first observed in the 24 hour or 48 hour image. Number of observations shown above each bar. p value calculated using Fisher's exact test comparing each bar to control.

(E) (left) Representative images of a chromatin bridge in Mitosis 0 that led to a binucleate, multipolar division in Mitosis 1. White arrowhead shows which daughter cell was photoconverted. Scale bar = 10 μm, inset scale bar = 5 μm. (right) Quantification of the frequency of bipolar and multipolar divisions in tetraploid cells that divided in Mitosis 1. Number of observations shown above bar.

n = normal mitosis, lc = lagging chromosome, cb = chromatin bridge, mn = micronuclei, mono = monopolar, binuc = binucleate

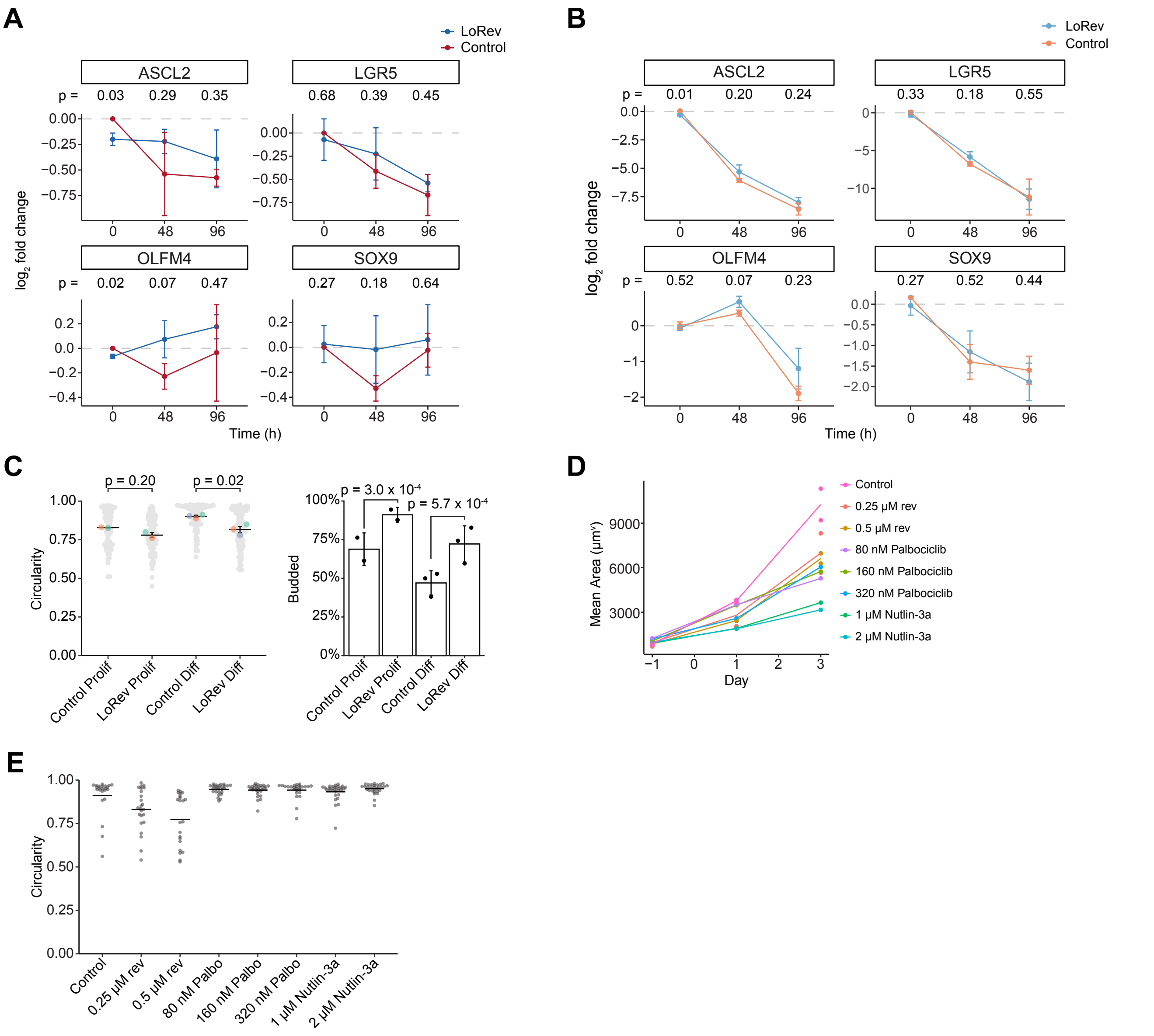

Supplemental Figure 5. Aneuploidy impairs intestinal stem cell differentiation

(A) Expression of stem cell markers measured by qPCR over time following treatment with vehicle-control (0.05% DMSO) or 0.25 μM reversine. DMSO and 0.25 μM reversine were washed out at time 0 hours and colonoids were grown in proliferation media for the remainder of the experiment. Points represent the mean of 3 biological replicates. Error bars represent standard deviation. p values were calculated using Welch's two sample t test.

(B) Expression of stem cell markers measured by qPCR over time following treatment with vehicle-control or 0.25 μM reversine. DMSO and 0.25 μM reversine were washed out at time 0 hours and colonoids were grown in differentiation media for the remainder of the experiment. Points represent the mean of 3 biological replicates. Error bars represent standard deviation. p values were calculated using Welch's two sample t test.

(C) (left) Quantification of circularity in colonoids treated with vehicle-control or 0.25 μM reversine then grown in drug-free proliferation or differentiation media with 96 hours. n = 3 biological replicates (right) Quantification of the frequency of colonoids that were budded after 96 hours in drug-free proliferation or differentiation media. colonoids were considered budded if their circularity was < 0.925. n = 3 biological replicates. p values were calculated using Fisher's exact test.

(D) Quantification of H2B-Dendra2 mean colonoid area over time. Drugs were added on Day -1 and drugs were washed out and differentiation media was added on Day 0. Line represents mean of 2 technical replicates.

(E) Quantification of circularity in colonoids treated with drugs shown for 24 hours then grown in differentiation media with 96 hours. n = 1 biological replicates. Each dot represents one colonoid. Line denotes mean circularity.
