# Supplementary material for: Simple aneuploidy evades p53 surveillance and promotes niche factor-independent growth in human intestinal organoids
Source: Mol Biol Cell. 2024 Jul 22;35(8):br15. doi: 10.1091/mbc.E24-04-0166 (PMC11321050; doi:10.1091/mbc.E24-04-0166)
Supplement: Supplementary file 3 [file reviewer_reportE24-04-0166.pdf]

Simple aneuploidy evades p53 surveillance and promotes niche factor-independent growth in human intestinal organoids

Blake Johnson, Albert Liu, Tianhao Bi, Yi Dong, Taibo Li, Dingjingyu Zhou, Akshay Narkar, Yufei Wu, Sean Sun, Tatianna Larman, Jin Zhu, and Rong Li

Corresponding author(s): Rong Li, National University of Singapore

Review Timeline:

Submission Date:	2024-04-14
Editorial Decision:	2024-05-08
Revision Received:	2024-06-19
Accepted:	2024-06-25

Editor-in-Chief: Matthew Welch

Transaction Report:

(Note: With the exception of the correction of typographical or spelling errors that could be a source of ambiguity, letters and reports are not edited. The original formatting of letters and referee reports may not be reflected in this compilation.)

RE: Manuscript #E24-04-0166
TITLE: Simple aneuploidy evades p53 surveillance and promotes niche factor-independent growth in human intestinal organoids

Dear Rong,

Thank you again for this interesting submission to the Molecular Biology of the Cell. As you can see the two reviewers are largely in favor of accepting this work for publication. Reviewer 1 had some questions about photoactivation. Reviewer 2 had some suggestions on what to emphasize, as well as some questions about the Reversine experiments.

I look forward to a revised manuscript that addresses these key reviewers comments.

Best,
Kerry Bloom
Monitoring Editor
Molecular Biology of the Cell

Dear Dr. Li,

The review of your manuscript, referenced above, is now complete. The Monitoring Editor has decided that your manuscript is not acceptable for publication at this time, but may be deemed acceptable after specific revisions are made, as described in the Monitoring Editor's decision letter above and the reviewer comments below.

A reminder: Please do not contact the Monitoring Editor directly regarding your manuscript. If you have any questions regarding the review process or the decision, please contact the MBoC Editorial Office (mboc@ascb.org).

When submitting your revision include a rebuttal letter that details, point-by-point, how the Monitoring Editor's and reviewers' comments have been addressed. (The file type for this letter must be "rebuttal letter"; do not include your response to the Monitoring Editor and reviewers in a "cover letter.") Please bear in mind that your rebuttal letter will be published with your paper if it is accepted, unless you have opted out of publishing the review history.

Authors are allowed 90 days to submit a revision. If this time period is inadequate, please contact us at mboc@ascb.org.

Revised manuscripts are assigned to the original Monitoring Editor whenever possible. However, special circumstances may preclude this. Also, revised manuscripts are often sent out for re-review, usually to the original reviewers when possible. The Monitoring Editor may solicit additional reviews if it is deemed necessary to render a completely informed decision.

In preparing your revised manuscript, please follow the instruction in the Information for Authors (www.molbiolcell.org/info-for-authors). In particular, to prepare for the possible acceptance of your revised manuscript, submit final, publication-quality figures with your revision as described.

To submit the rebuttal letter, revised manuscript, and figures, use this link: Link Not Available

MBoC offers the option to publish your work with immediate open access. Open access can increase the discoverability and usability of your research. If you would like to publish your paper with immediate open access but did not select this option during initial submission, please contact the editorial office at mbc@ascb.org.

Thank you for submitting your manuscript to MBoC. We look forward to receiving your revised paper.

Sincerely,

Eric Baker
Journal Production Manager
MBoC Editorial Office
mbc@ascb.org

Reviewer #1 (Remarks to the Author):

A critical question in cancer is how aneuploidy triggers a p53 response. It is clear that almost all highly aneuploid tumors lose TP53 activity but many tumors with lower amounts of aneuploid chromosomes have a p53 response. A recent important paper from the Li lab demonstrated that mouse colon organoids and human breast organoids can tolerate low amounts or aneuploidy much better than tissue culture cells. This suggests that the amount of aneuploidy that triggers TP53 is context dependent.

The manuscript from Johnson extends this paradigm by investigating human colon organoids. They generate aneuploidy by drug treatments (mostly MPS1 inhibition) and measure the consequences by a number of assays. Similar to mouse organoids the cells continue to proliferate after missegregation of a few chromosomes but arrest in a TP53 dependent manner after larger numbers of chromosomes are missegregated. They also provide demonstrate that the colonoids with simple aneuploidy are deficient in differentiating after withdrawal of stem cell factors providing a mechanism by which simple aneuploidy could promote transformation.

I mentioned that a similar response to p53 to simple and complex aneuploid phenotypes was recently demonstrated by the Li lab in other organoid systems. In addition, the concept that aneuploidy impairs stem cell differentiation has been established by others. Thus, the experiments are largely confirmatory, however, they are still important since human colon stem cells are the cell of origin for colon cancer. Moreover, there are important differences in mice and human colon cancer development, for example, APC mutations lead to small intestinal tumors in mice (unless they are combined with CIN). I also found the paper compelling by the use of some elegant approaches, for example, photoactivation of cells missegregating chromosomes to follow single cells within colonoids. Overall I support publication as long as the authors can adequately address my concerns.

Specific Concerns
-A key experiment is the photoactivation data in figure 3C and E. I have two questions about these data.

1). Why are most of the cells generating chromosome bridges? A simple spindle checkpoint inhibitor is expected to give lagging chromosomes as unattached or monotelic chromosomes would enter anaphase. This mechanism is supported by the single cell sequencing data in figure 2C, which shows whole chromosome changes, not chromosome breaks as would be expected from chromosome bridges. The authors should rule out the caveat that the imaging is generating phototoxicity that is damaging DNA and cannot be fixed during/after MPS1 inhibition and thus these experiments are not quantifying the same types of events as the other figures.

2) The cells displaying lagging chromosomes decreased numbers of proliferating cells and eliminated highly proliferative cells (5+) but the changes aren't statistically significant. The n for these experiments is low (13 events for lagging chromosomes in E) and it is unclear if they simply quantified more events that it would become significant. How did they determine that they had measured enough cells to conclude that there is no change? If they only take into consideration cells that scored zero in 3E, which are presumably cells that fully arrested, is there a change?

Reviewer #2 (Remarks to the Author):

This study investigates how induction of simple (1-3 chromosomes) vs complex (>4 chromosomes) aneuploidies impacts p53 pathway activation, cell cycle progression, and stem cell differentiation in primary colonic cells grown as colonoids. The authors assert that simple aneuploidies in colonoids are not sufficient to activate the p53 pathway and impair cellular proliferation (as opposed to increasingly complex aneuploidies that do correlate with increased p53 activity). The authors further observe that cells harboring simple aneuploidies exhibit sustained proliferation in differentiation medium lacking niche factors, thus implying that simple aneuploidies may delay/impair differentiation and potentiate the number of colonic stem cells that ultimately give rise to cancer.

Overall, this is a relatively clear and straightforward study that uses sophisticated approaches to address the experimental questions being posed. A weakness is that none of the authors' conclusions are particularly new (i.e. there are already numerous studies addressing aneuploidy/p53 and aneuploidy/differentiation links, which were cited). However, it is important to note that previous studies related to aneuploidy/p53/differentiation have yielded results that are variable and/or context- or tissue-type specific. Thus, I do believe this work in primary colonic organoids represents a valuable contribution to the field that should be published if it is completed in a rigorous manner, and I believe MBoC is an appropriate venue.

There are a few areas where the current manuscript needs revisions/clarifications before it can be published. These revisions are important, especially in light of the fact that many of the reported results are relatively subtle and border on significance. One primary area of concern is the work linking simple aneuplodies with p53 activation. The karyotyping and CENPA foci quantitations are very error prone and not good enough to reliably determine simple aneuploidies to infer consequences on p53 activation. These methods overestimate the amount of simple aneuploidies that are actually present, and the resulting data will confuse readers (specific examples detailed below). These data need to be clarified and more emphasis needs to be put on the more accurate scRNA-seq analyses of aneuploidy. The manuscript would also be strengthened by validating the results

(especially the differentiation result) by inducing aneuploidy in a manner different from Reversine drug treatment.

Major Points:
1. Based on their data, the authors conclude that simple aneuploidies in colonic epithelial cells in growing in organoids do not activate the p53 pathway and such aneuploid continue to proliferate. They write that "simple aneuploid cells generated with LoRev treatment did not activate p53 activation and continued to proliferate." However, I think such statements need to softened, as I don't believe they are fully supported by the current data. First, the authors write that "LoRev colonoids did not demonstrate increased abundance of p53 or p21, whereas both proteins were increased in HiRev colonoids (Figure 1C and S1F)." I do not agree with this assertion based on the date presented. Figure S1F appears to show clear p53 and p21 induction in cells treated with only 0.25 uM Reversine relative to controls (and the western blot does not seem to jive with the quantifications shown). Second, GSEA shows statistically significant p53 target gene enrichment in simple aneuploids (Fig. 2h). Third, there is trending decrease in Edu incorporation in low Reversine cells (Fig. 1D and Fig. 4D). I think these data imply that simple aneuploidies do impact the p53 pathway and can have effects on proliferation, and that this effect is then potentiated with increasing aneuploidy. The way the manuscript is written now makes it seem as though simple aneuplodies have no effect on p53/proliferation.

2. Using scRNA-seq data to infer aneuploidy is clearly the most reliable method used in this manuscript and these data should be more heavily emphasized. The foundational data currently presented in Figure 1, which relies on karyotyping to infer simple vs complex aneuplodies, is clearly very error prone and difficult to interpret. In that experiment, normal controls show 25% of cells with simple aneuploidies, which we know is not true and represents an artefact of counting chromosomes in spreads. Moreover, acquiring such karyotyping data requires that cells progress through the cell cycle and enter mitosis in order to even be assessed, which would thus select only those cells capable of proliferating for karyotyping analysis (which would bias aneuploidy estimates). I think the authors should lead with the stronger scRNA-seq data to validate the effects of the drugs. While I think the karyotyping experiments still have value, it is important for the authors to at least detail the limitations of that approach.

3. It is imperative that the authors show that higher doses of Reversine do not activate p53 by promoting DNA damage.

4. Experiments would benefit from additional methods to induce aneuploidy, rather than on reliance of Reversine only (particularly in Figure 4).

5. In Figures 2i and 2j, the authors make the claim that increased aneuploidy causes increased p53 expression. In 2i, where they attempt to correlate the number of aneuploid chromosomes with the p53 pathway score, the R^2 value is 0.4. While there does seem to be an increase in p53 pathway score from euploid to simple aneuploid, there doesn't seem to be a significant difference between simple and complex aneuploid cells. Can these be broken up into two parts?

6. Fig 2J shows correlation of chromosome number with p53 activation. The data shown here imply that aneuploidy caused by chromosome losses (less than 46 chromosomes per cell) decreases p53 activation. This contradicts their earlier points that complex aneuploidy leads to p53 activation. I don't believe the authors mean to make this argument, it simply looks that way because of the variability in assessing chromosome numbers by CENPA foci, which is very error prone. This needs to be clarified to avoid confusing the reader. Additionally, the R^2 value for this plot is a weak 0.09, with the positive slope likely driven by the 2 cells with vastly increased chromosome number.

7. Figure 4C, should be unpaired test like in 4G. Still significant?

8. I have concerns that all of the cell proliferation/differentiation assays in Figure 4 are performed on the bulk population level after low dose Reversine treatment (where only 50% cells develop aneuploidy), so it can't be fully determined if there is a causal role of aneuploidy on stem cell potential. This claim would be strengthened by isolating "stem cell like cells" from bud/crypts (EdU+ or ASCL2 reporter positive) and comparing karyotypes/transcriptomes/organoid formation potential from single cells to cells isolated from non-bud/crypt areas of organoids after Reversine treatment. Also, why not also perform these experiments with high dose Reversine to further determine if level of aneuploidy correlates with decreased stem cell capacity to differentiate?

Minor:

9. Figure 1A. What is the point of showing all of the drugs that cause aneuploidy if there is no follow up? It makes for a distracting figure.

10. Figure 1C. Western blot needs to be shown in main figure.

11. Figure 3C. It is important to see what percentage of cells divide without photoconversion (non-photoconverted control) to rule out that photoconversion alone does not induce stress or DNA damage that may hinder proliferation (>30% of control cells never enter mitosis).

12. Figure 3D. It would be informative to look at fates of both daughter cells in tracking experiments because they will be equally aneuploid (i.e. if one daughter cells gains 3 chromosomes, the other would lose three). Are fates similar between daughters? If not, how could that be explained? This result might suggest that factors other than aneuploidy are contributing to cell fate.

13. Figure 3C-E claims that there is no statistically significant difference in mitotic fate in cells that undergo normal mitosis vs lagging chromosome mitosis. However, lagging chromosomes also promote increased cell death at both 24 and 48 hours after reversine (Fig S3B). Since most time points analyzed are at 24 hours and later, results may be biased towards surviving cells that may have lower p53 functionality to begin with. This needs to be clarified.

14. Figure 4F only two biological replicates.

15. In 3e, the authors say that chromosome bridge rarely result in further divisions, but in 3c, half of them are able to divide after the first chromosome bridge. Does this mean that there isn't enough aneuploidy after the first division for p53 activation to occur, but after the second division there is enough aneuploidy? Further clarification is needed.

16. The authors distinguish lagging and bridging chromosomes when assessing fates, but why not distinguish tetraploidy and MN as well? Both are conspicuous features and are known to induce p53 through either DNA damage (generated in MN) or from inhibition of MDM2 (due to extra centrosomes and PIDD/Hippo activation).

17. The authors write, "Although LoRev colonoids did not have significantly reduced the total expression of Muc2, a marker for differentiated goblet cells, Muc2-positive cells were not found within proliferative buds, further supporting that these buds are stem cell niches (Figure 4G)." These data need to be shown more clearly and quantified specifically in buds.

18. The authors write that "LoRev led to aneuploidy predominately by causing lagging chromosomes". This is somewhat misleading as Cimini et al, 2004 and Thompson et al,. 2011 showed that lagging chromosomes often do not missegregate. Missegregated chromosomes typically come from non-lagging chromosomes (presumably unattached or mono-oriented). Thus, while lagging chromosomes are often a good indicator that chromosome missegregations rates are elevated, the lagging chromosome per se is not missegregated.

Response to reviewers

We thank the reviewers for their thorough and constructive suggestions for improving our manuscript. In the following responses, we have addressed the reviewers' concerns to the best of our ability. We appreciate the reviewers' recognition of the importance of this work and feel their suggestions have helped us improve the clarity and rigor of our findings. We have included new data showing no evidence of DNA damage in HiRev colonoids, and impaired differentiation of aneuploid cells generated by a second drug. Reviewer comments are in blue text and our responses are in black.

Reviewer #1 (Remarks to the Author):

A critical question in cancer is how aneuploidy triggers a p53 response. It is clear that almost all highly aneuploid tumors lose TP53 activity but many tumors with lower amounts of aneuploid chromosomes have a p53 response. A recent important paper from the Li lab demonstrated that mouse colon organoids and human breast organoids can tolerate low amounts or aneuploidy much better than tissue culture cells. This suggests that the amount of aneuploidy that triggers TP53 is context dependent.

The manuscript from Johnson extends this paradigm by investigating human colon organoids. They generate aneuploidy by drug treatments (mostly MPS1 inhibition) and measure the consequences by a number of assays. Similar to mouse organoids the cells continue to proliferate after missegregation of a few chromosomes but arrest in a TP53 dependent manner after larger numbers of chromosomes are missegregated. They also provide demonstrate that the colonoids with simple aneuploidy are deficient in differentiating after withdrawal of stem cell factors providing a mechanism by which simple aneuploidy could promote transformation.

I mentioned that a similar response to p53 to simple and complex aneuploid phenotypes was recently demonstrated by the Li lab in other organoid systems. In addition, the concept that aneuploidy impairs stem cell differentiation has been established by others. Thus, the experiments are largely confirmatory, however, they are still important since human colon stem cells are the cell of origin for colon cancer. Moreover, there are important differences in mice and human colon cancer development, for example, APC mutations lead to small intestinal tumors in mice (unless they are combined with CIN). I also found the paper compelling by the use of some elegant approaches, for example, photoactivation of cells missegregating chromosomes to follow single cells within colonoids. Overall I support publication as long as the authors can adequately address my concerns.

We thank the reviewer for their comments and have addressed each individually below.

Specific Concerns
-A key experiment is the photoactivation data in figure 3C and E. I have two questions about these data.

1). Why are most of the cells generating chromosome bridges? A simple spindle checkpoint inhibitor is expected to give lagging chromosomes as unattached or monotelic chromosomes would enter anaphase. This mechanism is supported by the single cell sequencing data in figure 2C, which shows whole chromosome changes, not chromosome breaks as would be expected from chromosome bridges. The authors should rule out the caveat that the imaging is generating phototoxicity that is damaging DNA and cannot be fixed during/after MPS1 inhibition and thus these experiments are not quantifying the same types of events as the other figures.

The reviewer is correct that chromatin bridges were more frequently observed than lagging chromosomes in the photoconversion experiments. We agree that MPS1 inhibition is typically associated with unattached or monotelic chromosomes leading to lagging chromosomes. On the other hand, DNA damage can lead to chromatin bridges (and vice versa). However, we observed that 0.5 µM reversine shortened the duration of mitosis (Figure S1D). Since sister chromatid decatenation continues into mitosis, MPS1 inhibition can lead to chromatin bridges without DNA damage if anaphase progresses prior to the completion of decatenation (PMID: 24065128) and chromatin bridges have been observed in other studies using MPS1 inhibitors (PMID: 34819364).

It is also important to note that our single cell sequencing analysis was focused on identifying whole chromosome changes, not segmental variation. Although analysis of segmental gains or losses is possible with this dataset, additional computational optimization beyond the scope of this manuscript would be necessary. We have included this caveat in the revised manuscript: “It is important to note that our analysis was designed to identify whole chromosome, not segmental, changes in copy number” (lines 128-130).

Regarding the possibility that imaging led to DNA damage that promotes chromatin bridges, we imaged colonoids expressing the DNA damage reporter 53BP1-mCherry using similar imaging conditions to those used to image Mitosis 0 in the photoconversion experiments. We did not observe increased 53BP1 foci following 4 hours of imaging (the amount of time that colonoids were imaged during Mitosis 0 in Figure 3) with or without reversine treatment (Figure S4B). Additionally, we observed chromatin bridges in HiRev cells with no evidence of DNA damage foci prior to entering mitosis (Figure S4B). As discussed in lines 188-191, this suggests that imaging-related phototoxicity did not contribute to chromatin bridge formation. We feel that, regardless of the underlying mechanism leading to chromatin bridges, the observation of chromosome bridges does not affect our proposal that aneuploid cells, particularly those arising from division with lagging chromosomes, can continue to proliferate.

2) The cells displaying lagging chromosomes decreased numbers of proliferating cells and eliminated highly proliferative cells (5+) but the changes aren't statistically significant. The n for these experiments is low (13 events for lagging chromosomes in E) and it is unclear if they simply quantified more events that it would become significant. How did they determine that they had measured enough cells to conclude that there is no change? If they only take into consideration cells that scored zero in 3E, which are presumably cells that fully arrested, is there a change?

We thank the reviewer for their comment. We found that the displayed total number of tracked cells was erroneously carried over from an earlier version of the figure. Figure 3E has now been updated with the correct number of tracked cells above each bar, including 25 in the lagging chromosomes category. The original figure displayed the correct proportion for each number of progeny and the correct statistical tests. We apologize for this error and have double-checked all other figures to ensure similar errors are not present elsewhere.

Here, we hoped to directly determine if cells could continue proliferating following chromosome mis-segregation as suggested in Figures 1 and 2. Due to the highly time-consuming nature of this experiment, we felt the number of observations was sufficient to conclude that at least some cells do continue to divide after mitotic error. Based on the reviewer's suggestion, we compared cells with 0 and 1 progeny between groups. We have clarified in lines 207-209 that cells with 0 progeny likely died as the original photoconverted daughter was no longer present, and cells with 1 progeny, likely representing the original photoconverted cell, fully arrested. We see that daughters from divisions with lagging chromosomes are more likely to have 0 or 1 progeny suggesting more of these cells die or arrest than daughters from normal divisions (Figure 3E). This is consistent with the trend toward increased cell death following lagging chromosome in Mitosis 0 shown in Figure S4D. “These observations suggest that many, but not all, cells continuing to proliferate following chromosome mis-segregation.” (lines 216-218).

Reviewer #2 (Remarks to the Author):

This study investigates how induction of simple (1-3 chromosomes) vs complex (>4 chromosomes) aneuploidies impacts p53 pathway activation, cell cycle progression, and stem cell differentiation in primary colonic cells grown as colonoids. The authors assert that simple aneuploidies in colonoids are not sufficient to activate the p53 pathway and impair cellular proliferation (as opposed to increasingly complex aneuploidies that do correlate with increased p53 activity). The authors further observe that cells harboring simple aneuploidies exhibit sustained proliferation in differentiation medium lacking niche factors, thus implying that simple aneuploidies may delay/impair differentiation and potentiate the number of colonic stem cells that ultimately give rise to cancer.

Overall, this is a relatively clear and straightforward study that uses sophisticated approaches to address the experimental questions being posed. A weakness is that none of the authors' conclusions are particularly new (i.e. there are already numerous studies addressing aneuploidy/p53 and aneuploidy/differentiation links, which were cited). However, it is important to note that previous studies related to aneuploidy/p53/differentiation have yielded results that are variable and/or context- or tissue-type specific. Thus, I do believe this work in primary colonic organoids represents a valuable contribution to the field that should be published if it is completed in a rigorous manner, and I believe MBoC is an appropriate venue.

There are a few areas where the current manuscript needs revisions/clarifications before it can be published. These revisions are important, especially in light of the fact that many of the

reported results are relatively subtle and border on significance. One primary area of concern is the work linking simple aneuplodies with p53 activation. The karyotyping and CENPA foci quantitations are very error prone and not good enough to reliably determine simple aneuploidies to infer consequences on p53 activation. These methods overestimate the amount of simple aneuploidies that are actually present, and the resulting data will confuse readers (specific examples detailed below). These data need to be clarified and more emphasis needs to be put on the more accurate scRNA-seq analyses of aneuploidy. The manuscript would also be strengthened by validating the results (especially the differentiation result) by inducing aneuploidy in a manner different from Reversine drug treatment.

Major Points:

1. Based on their data, the authors conclude that simple aneuploidies in colonic epithelial cells in growing in organoids do not activate the p53 pathway and such aneuploid continue to proliferate. They write that "simple aneuploid cells generated with LoRev treatment did not activate p53 activation and continued to proliferate." However, I think such statements need to softened, as I don't believe they are fully supported by the current data. First, the authors write that "LoRev colonoids did not demonstrate increased abundance of p53 or p21, whereas both proteins were increased in HiRev colonoids (Figure 1C and S1F)." I do not agree with this assertion based on the date presented. Figure S1F appears to show clear p53 and p21 induction in cells treated with only 0.25 uM Reversine relative to controls (and the western blot does not seem to jive with the quantifications shown). Second, GSEA shows statistically significant p53 target gene enrichment in simple aneuploids (Fig. 2h). Third, there is trending decrease in Edu incorporation in low Reversine cells (Fig. 1D and Fig. 4D). I think these data imply that simple aneuploidies do impact the p53 pathway and can have effects on proliferation, and that this effect is then potentiated with increasing aneuploidy. The way the manuscript is written now makes it seem as though simple aneuplodies have no effect on p53/proliferation.

We have included replicate 1 of the western blots used for quantification in Figure 1C as Figure 1B, and replicates 2 and 3 as Figure S1G. The quantification shown in Figure 1C was obtained using an area under the curve strategy to quantify the total intensity of each band. p53 and p21 abundance is shown relative to loading control GAPDH. Biological replicates are normalized using sum normalization (PMID: 24475266). Based on the reviewer’s comment that the western blot image appears to disagree with the quantification, we used an alternative quantification strategy that examined the mean intensity of a defined area around each band (see figure below). Using this strategy, we still did not find a statistically significant difference in p53 or p21 abundance between control and LoRev in agreement with Figure 1C. We would also like to reiterate that Figure 1C is only quantifying and comparing p53 and p21 protein abundance 24 hrs after drug washout for each condition. However, the reviewer was correct that there does appear to be a trend toward increasing p53 in LoRev, and we cannot definitively rule out that LoRev has some effect on the p53 pathway. Based on the western blot and EdU labeling results and the scRNA seq data, and the findings in Figure 3E described in our response to Reviewer #1, comment #2, we agree with the reviewer’s point that we do not have sufficient evidence to conclude simple aneuploid cells have no effect on p53 activation and proliferation. Our data

allow us to conclude that some, but not all, simple aneuploid cells are able to escape p53-mediated cell cycle arrest and continue to divide. We have made the following text changes to represent this point more accurately (specific text changes highlighted in **bold** below):

(Line 31-33, abstract): “We found that simple aneuploidy, characterized by 1-3 gained or lost chromosomes, **resulted in little or modest** p53 activation and cell cycle arrest compared with more complex aneuploid cells.”

(Line 107-109): LoRev colonoids proliferated comparably to vehicle-control as showed by similar EdU incorporation, **although we did observe a trend toward reduced proliferation**.”

(Lines 114-115, conclusion of figure 1): “Together, these data suggest that **many, but perhaps not all,** simple aneuploid cells can evade p53 surveillance and continue to proliferate.”

(Lines 262-267): “In contrast, simple aneuploid cells generated with LoRev treatment had **less effect** on p53 activity. **Our data suggest that simple aneuploid cells can mildly activate the p53 pathway in human colonoids, particularly at the transcriptional level, and this effect is markedly enhanced as karyotype complexity increases.** Importantly, our data also suggest that **at least a subset of** aneuploid cells gaining or losing one or a few chromosomes can evade p53 surveillance and continue to proliferate.”

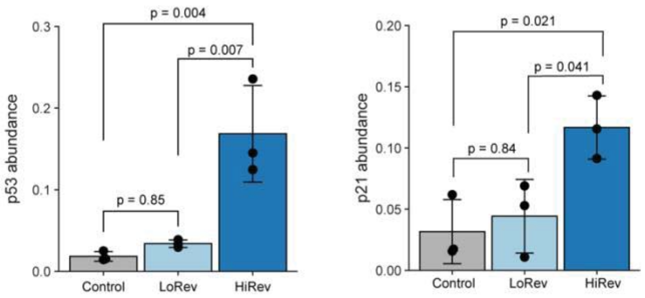

Quantification of the immunoblots in Figure 1C and S1G using mean intensity around each band. Relative abundance to GAPDH, loading control. n = 3 biological replicates. Biological replicates are normalized using sum normalization. Bars represent mean and error bars represent standard deviation. Abundances compared using ANOVA and post-hoc Tukey test. Tukey p values are shown.

2. Using scRNA-seq data to infer aneuploidy is clearly the most reliable method used in this manuscript and these data should be more heavily emphasized. The foundational data currently presented in Figure 1, which relies on karyotyping to infer simple vs complex aneuplodies, is clearly very error prone and difficult to interpret. In that experiment, normal controls show 25% of cells with simple aneuploidies, which we know is not true and represents an artefact of counting chromosomes in spreads. Moreover, acquiring such karyotyping data requires that cells progress through the cell cycle and enter mitosis in order to even be assessed, which would thus select only those cells capable of proliferating for karyotyping analysis (which would bias aneuploidy estimates). I think the authors should lead with the stronger scRNA-seq data to

validate the effects of the drugs. While I think the karyotyping experiments still have value, it is important for the authors to at least detail the limitations of that approach.

The current figure order reflects the true logical progression of our experiments, and we also feel that the manuscript flows better to maintain this original order. However, we agree with the reviewer that quantifying aneuploidy via metaphase spread karyotyping has limitations. We have included the following in lines 115-117: "Importantly, karyotyping by metaphase spreads is error prone and can only assess dividing cells, which could explain the unexpectedly high proportion of aneuploid cells in control colonoids." We found that metaphase spread karyotyping only resulted in undercounting chromosomes in control colonoids (Figure 1A). LoRev treatment increased the number of cells with both gain and loss of chromosomes. If we exclude loss of chromosomes, which we agree are more likely to represent technical artifact, we observe 0% aneuploidy in control colonoids and 39% in LoRev. We also observed mitotic errors in ~50% of divisions when colonoids were treated with LoRev (Figure S1F). Together, these data allow us to feel confident that LoRev does lead to simple aneuploidy in many cells within colonoids.

3. It is imperative that the authors show that higher doses of Reversine do not activate p53 by promoting DNA damage.

We have included an additional supplemental figure documenting our findings related to DNA damage in HiRev colonoids (Figure S3). We did not find evidence of DNA damage causing p53 activation in HiRev colonoids and have presented the finding on lines 162-181.

4. Experiments would benefit from additional methods to induce aneuploidy, rather than on reliance of Reversine only (particularly in Figure 4).

We appreciate this suggestion and have investigated the effect of treatment with 1 µM ZM-447439, an Aurora B kinase inhibitor, which resulted in similar karyotype complexity and heterogeneity as 0.25 µM reversine (LoRev) (Figure S1D). We looked at ASCL2 reporter activity under differentiation conditions. We found that ZM treatment result in increased ASCL2 activity comparable to that seen in LoRev (Figure 4F) suggesting that impaired differentiation is not specific to reversine or MPS1 kinase inhibition. We regret that, for practical reasons, we cannot repeat the entire study with another inhibitor. We feel that previous studies from us and others have established MPS1 kinase inhibition as a reliable method of inducing aneuploidy.

5. In Figures 2i and 2j, the authors make the claim that increased aneuploidy causes increased p53 expression. In 2i, where they attempt to correlate the number of aneuploid chromosomes with the p53 pathway score, the R^2 value is 0.4. While there does seem to be an increase in p53 pathway score from euploid to simple aneuploid, there doesn't seem to be a significant difference between simple and complex aneuploid cells. Can these be broken up into two parts?

We have compared the p53 pathway score between the 3 groups in our scRNA-seq data (euploid, simple aneuploid, and complex aneuploid) (Figure 2I). We found that the p53 pathway score was increased in complex aneuploid cells compared with simple aneuploid cells. We also

found that the p53 pathway score was higher in simple aneuploid cells than euploid cells. This is in agreement with the comment from Reviewer #2 that simple aneuploid does have some effect on p53 activity, which is reflected in our text changes as described in the response to this reviewer's comment #1.

6. Fig 2J shows correlation of chromosome number with p53 activation. The data shown here imply that aneuploidy caused by chromosome losses (less than 46 chromosomes per cell) decreases p53 activation. This contradicts their earlier points that complex aneuploidy leads to p53 activation. I don't believe the authors mean to make this argument, it simply looks that way because of the variability in assessing chromosome numbers by CENPA foci, which is very error prone. This needs to be clarified to avoid confusing the reader. Additionally, the R^2 value for this plot is a weak 0.09, with the positive slope likely driven by the 2 cells with vastly increased chromosome number.

We thank the reviewer for suggesting this important clarification. Indeed, we did not mean to suggest chromosome loss is associated with decreased p53 pathway activity. Instead of looking at the relationship between absolute chromosome number and p53 intensity, we have compared p53 intensity between euploid, simple, and complex aneuploid cells in a similar manner to Figure 2I (Figure 2K). For this analysis, all cells with ≤47 chromosomes were considered euploid because of the difficulty of accurately identifying simple aneuploid cells using this method. We found that complex aneuploid cells had increased p53 activity when compared with control, and found no significant difference when comparing control and simple aneuploid or simple and complex aneuploid. This is now clearly described in lines 149-159.

7. Figure 4C, should be unpaired test like in 4G. Still significant?

We have now done an unpaired t test. The groups remain statistically significantly different ($p = 0.045$). Since we have now included 3 groups in the figure (HiRev was added), we performed an ANOVA with post hoc Tukey test. The Tukey p values are shown in the figure.

8. I have concerns that all of the cell proliferation/differentiation assays in Figure 4 are performed on the bulk population level after low dose Reversine treatment (where only 50% cells develop aneuploidy), so it can't be fully determined if there is a causal role of aneuploidy on stem cell potential. This claim would be strengthened by isolating "stem cell like cells" from bud/crypts (EdU+ or ASCL2 reporter positive) and comparing karyotypes/transcriptomes/organoid formation potential from single cells to cells isolated from non-bud/crypt areas of organoids after Reversine treatment. Also, why not also perform these experiments with high dose Reversine to further determine if level of aneuploidy correlates with decreased stem cell capacity to differentiate?

We agree with the reviewer that future single cell analyses will be crucial for definitively establishing a causal link between aneuploidy and impaired differentiation. We have included this in our discussion: "Our analysis examined only bulk populations including both simple aneuploid and euploid cells. Isolating single proliferate cells under differentiation conditions and

studying their karyotype and stem cell potential will help establish a causal role for aneuploidy in impairing colon stem cell differentiation" (lines 283-287).

We have also included data on the effect of HiRev on differentiation in Figures 4C and 4E. While HiRev did not result in statistically significantly greater impairment of differentiation compared with LoRev, there was a trend in this direction suggesting more aneuploidy may lead to more cells retaining stem cell capacity. We have mentioned this result in lines 239-240.

Minor:

9. Figure 1A. What is the point of showing all of the drugs that cause aneuploidy if there is no follow up? It makes for a distracting figure.

During our initial investigation we chose to explore numerous strategies for inducing aneuploid populations of varying complexity and heterogeneity in human colonoids. We felt that this could be a useful resource for investigators interested in generating aneuploid colonoids in the future. However, we agree with the reviewer that this data was distracting in the main figure and have moved it to Figure S1D.

10. Figure 1C. Western blot needs to be shown in main figure.

We have included the blot image in Figure 1B and the remaining replicates in Figure S1G.

11. Figure 3C. It is important to see what percentage of cells divide without photoconversion (non-photoconverted control) to rule out that photoconversion alone does not induce stress or DNA damage that may hinder proliferation (>30% of control cells never enter mitosis).

Photoconversion in these experiments was necessary because it was difficult to track cells over such an extended period of time within organoids without unique labeling (provided by photoconversion). The average cell cycle length for colonoids was about 16 hours (PMID: 33761356), so 70% of cells dividing over the 24 h period was close to expected. Furthermore, the proliferation rate without photoconversion would be important if we had concluded that aneuploidy was preventing proliferation. However, our conclusion is that some aneuploids can continue to divide, and thus the concern that photoconversion itself is hindering proliferation is less relevant.

12. Figure 3D. It would be informative to look at fates of both daughter cells in tracking experiments because they will be equally aneuploid (i.e. if one daughter cells gains 3 chromosomes, the other would lose three). Are fates similar between daughters? If not, how could that be explained? This result might suggest that factors other than aneuploidy are contributing to cell fate.

We agree that comparing the fate of both daughter cells would be interesting. We elected to only photoconvert 1 daughter cell to avoid any ambiguity about the origin of photoconverted

cells that we observed. Tracking both daughters using an improved technique could be very informative to future studies investigating differences in responses between gaining and losing chromosomes, which we believe still remains a very interesting underexplored area with regards to aneuploid cell fate.

13. Figure 3C-E claims that there is no statistically significant difference in mitotic fate in cells that undergo normal mitosis vs lagging chromosome mitosis. However, lagging chromosomes also promote increased cell death at both 24 and 48 hours after reversine (Fig S3B). Since most time points analyzed are at 24 hours and later, results may be biased towards surviving cells that may have lower p53 functionality to begin with. This needs to be clarified.

Cells that died are included in the analysis in Figure 3E as 0 progeny, to avoid biasing toward surviving cells. We specifically compared the number of photoconverted cells with 0 (died) or 1 (arrested) progeny between groups in Figure 3E per the suggestion of Reviewer #1. We find that lagging chromosomes lead to increased cells with 0 or 1 progeny. We concluded that there are instances in which chromosome missegregation that likely leads to simple aneuploidy causes cell cycle arrest or death. This is in line with the Reviewer #2’s first point that simple aneuploids can affect the p53 pathway in some way. We have discussed this in lines 207-209.

14. Figure 4F only two biological replicates.

We performed this experiment with additional biological replicates. This new data is shown in the updated Figure 4F.

15. In 3e, the authors say that chromosome bridge rarely result in further divisions, but in 3c, half of them are able to divide after the first chromosome bridge. Does this mean that there isn't enough aneuploidy after the first division for p53 activation to occur, but after the second division there is enough aneuploidy? Further clarification is needed.

We indeed observed that binucleate cells that arose from chromatin bridges often underwent multipolar division, which likely resulted in highly aneuploid cells, leading to p53 activation after the second mitosis. We have clarified this point as explained above on lines 213-216.

16. The authors distinguish lagging and bridging chromosomes when assessing fates, but why not distinguish tetraploidy and MN as well? Both are conspicuous features and are known to induce p53 through either DNA damage (generated in MN) or from inhibition of MDM2 (due to extra centrosomes and PIDD/Hippo activation).

We thank the reviewer for this suggestion. We examined daughter cells from divisions with lagging chromosomes in Mitosis 0 and found that “the presence or absence of a micronucleus did not impact the division frequency” (lines 192-193). The data is shown in Figure S4A.

While the fate of tetraploid cells is an important question, we have decided to focus on aneuploidy in this study and have chosen to not include further investigation of tetraploidy in the manuscript.

17. The authors write, "Although LoRev colonoids did not have significantly reduced the total expression of Muc2, a marker for differentiated goblet cells, Muc2-positive cells were not found within proliferative buds, further supporting that these buds are stem cell niches (Figure 4G)." These data need to be shown more clearly and quantified specifically in buds.

We agree with the reviewer that this statement would require additional evidence. We have modified it to read “LoRev colonoids had a trend toward reduced expression of Muc2, a marker for differentiated goblet cells, but this difference was not statistically significant” (lines 247-249).

18. The authors write that "LoRev led to aneuploidy predominately by causing lagging chromosomes". This is somewhat misleading as Cimini et al, 2004 and Thompson et al,. 2011 showed that lagging chromosomes often do not missegregate. Missegregated chromosomes typically come from non-lagging chromosomes (presumably unattached or mono-oriented). Thus, while lagging chromosomes are often a good indicator that chromosome missegregations rates are elevated, the lagging chromosome per se is not missegregated.

We have modified this statement to read “Lagging chromosomes were the predominant mitotic abnormalities observed in LoRev colonoids” (lines 97-98).

RE: Manuscript #E24-04-0166R
TITLE: "Simple aneuploidy evades p53 surveillance and promotes niche factor-independent growth in human intestinal organoids"

Dear Rong and colleagues,

Thank you very much for your detailed response to the reviewers critiques. I am very pleased to inform you that your manuscript is now suitable for publication in the Molecular Biology of the Cell. Thank you for your submission of these interesting results.

Best,
Kerry

Kerry Bloom
Monitoring Editor
Molecular Biology of the Cell

Dear Dr. Li:

Congratulations on the acceptance of your manuscript! Thank you for choosing to publish your work in Molecular Biology of the Cell (MBoC).

Within 10 days, an unedited PDF of your manuscript will be published on MBoC in Press, an early release version of the journal. The date your manuscript appears on this site is the official publication date.

Your copyedited and typeset manuscript will be scheduled for publication in the next available issue of MBoC. Our production team will notify you when the page proofs of your paper are ready for your review.

MBoC offers the option to publish your paper with immediate open access. Open access can increase the discoverability and usability of your research. If you would like to publish your paper with immediate open access but did not select this option during initial submission, please contact the MBoC Editorial Office (mbc@ascb.org).

We invite you to submit images related to your accepted manuscript to be considered for the journal cover.
Cover images must contain color, TIF Format
Size image at 7.4" wide x 5.85" deep
RGB and CMYK color (please submit two separate files, one in each format)
300 dpi resolution

Please also send a 1-paragraph description of the image that a general audience could understand. Include with the caption the name and institution of the individual(s) the image should be attributed to. Please provide this description in a Word file. You can send the file via email or by your favorite file transfer service to mboc@ascb.org.

Authors of Articles and Brief Communications are also encouraged to create a short video abstract to accompany their article when it is published. These video abstracts, known as Science Sketches, are up to 2 minutes long and will be published on YouTube and then embedded in the article abstract. Science Sketch Editors on the MBoC Editorial Board will guide you as you prepare your video. Information about how to prepare and submit a video abstract is available at www.molbiolcell.org/science-sketches. Please contact mboc@ascb.org if you are interested in creating a Science Sketch.

We look forward to publishing your paper in MBoC.
Sign up for MBoC's new content alerts here https://tinyurl.com/3bcsyk3e

Sincerely,

Eric Baker
Journal Production Manager
MBoC Editorial Office

mbc@ascb.org
